# Electron Transport in Carbon Nanotubes with Adsorbed Chromium Impurities

**DOI:** 10.3390/ma12030524

**Published:** 2019-02-10

**Authors:** Stanislav Repetsky, Iryna Vyshyvana, Yasuhiro Nakazawa, Sergei Kruchinin, Stefano Bellucci

**Affiliations:** 1 Institute of High Technologies, Taras Shevchenko Kyiv National University, 02033 Kyiv, Ukraine; srepetsky0208@gmail.com (S.R.); i.vyshyvana@gmail.com (I.V.); 2 Department of Chemistry, Graduate School of Science, Osaka University, 560-0043 Osaka, Japan; nakazawa@chem.sci.osaka-u.ac.jp; 3 Bogolyubov Institute for Theoretical Physics, 03143 Kyiv, Ukraine; sergeikruchinin@yahoo.com; 4 INFN-Laboratori Nazionali di Frascati, Via E. Fermi 40, 00044 Frascati, Italy

**Keywords:** carbon nanotubes, chromium impurities, spin-depended transport, Green’s function, multiband Hamiltonian, electron correlation, 71.15.Dx, 31.15.E-, 31.15.A-, 71.15.Nc

## Abstract

We employ Green’s function method for describing multiband models with magnetic impurities and apply the formalism to the problem of chromium impurities adsorbed onto a carbon nanotube. Density functional theory is used to determine the bandstructure, which is then fit to a tight-binding model to allow for the subsequent Green’s function description. Electron–electron interactions, electron–phonon coupling, and disorder scattering are all taken into account (perturbatively) with a theory that involves a cluster extension of the coherent potential approximation. We show how increasing the cluster size produces more accurate results and how the final calculations converge as a function of the cluster size. We examine the spin-polarized electrical current on the nanotube generated by the magnetic impurities adsorbed onto the nanotube surface. The spin polarization increases with both increasing concentration of chromium impurities and with increasing magnetic field. Its origin arises from the strong electron correlations generated by the Cr impurities.

## 1. Introduction

The theory for disordered materials is less well developed than the theory for periodic systems. The simplest theory of a disordered system comes from the Born approximation to scattering theory for particles moving in the periodic system with isolated scatterers due to the disorder. However, this is a weak-coupling theory which works well only when the scatterer is similar to the host. This will certainly not be the case with transition metal or rare-earth impurities in conventional sp-metals. Alternatives, such as methods based on pseudopotentials [1] often fail because the nonlocal nature of the pseudopotential makes them difficult to transfer from one environment to another. This situation has changed, to some degree, with the introduction of Vanderbilt’s ultrasoft pseudopotential [2,3] and the use of projector augmented waves in density functional theory, as proposed by Blohl [4,5]. This approach was further developed via the generalized gradient approximation (GGA) to density functional theory of multi-electron systems in a series of papers by Perdew and co-workers [6,7,8,9,10]. The wave function for valence electron states (called an all-electron orbital) is modified (within the projector augmented-wave approach) to a pseudo-orbital, with a pseudo-orbital expanded in plane waves. The pseudo-orbital is identical to the true core state outside the core region and is smoothly extended inside the core. The pseudo-orbitals are expanded in pseudo partial waves and are represented via a radial function times spherical harmonics (in the augmentation region). The same coefficients are employed for the all-electron orbitals, when expanded via partial waves that are described by the Kohn–Sham equation. The expression for the effective Hamiltonian operator, which is used as a Schroedinger equation for the pseudo-orbital, is derived by minimizing the total energy functional [10]. Using this equation, and expanding the pseudo-orbital by plane waves, we derive a set of equations for the expansion coefficients. From this system, one finds the electron bandstructure, the wave functions, and the value of the total energy functional. Calculations are performed within the VASP program package [10,11]. Adapting cluster methods with the GAUSSIAN program package [10,12], this approach can be employed for molecular electronic structure determination.

This strategy has recently been applied to solve a number of different periodic problems or problems with large molecules [13,14,15,16,17,18,19]. It is based on the tight-binding model and density functional theory, which includes long-range coulomb interactions of electrons on different sites of crystal lattice. The long-range coulomb interaction of electrons on different sites is described in the local density approximation.

So far, these methods [6,7,8,9,10,13,14,15,16,17,18,19] have only been applied to periodic systems. In disordered crystals, the effects associated with localized electronic states and lattice vibrations are also important. They cannot be described by the aforementioned approaches. Different strategies need to be developed.

This is, of course, an old problem. The simplest treatment of it comes from tight-binding-model approaches coupled with multiple-scattering theory to yield the coherent potential approximation. Here, Slater and Koster [20,21] laid the groundwork for tight-binding model descriptions of periodic crystals, and later this approach was generalized to the case of disordered systems [22,23].

This method for describing magnetic alloys begins with the effective potential in the Kohn–Sham equation [24,25], which consists of the atomic charge potential and a Pauli term, which is expressed through the magnetic field induction. The atomic potential and the magnetic field induction are found through variational derivatives of the exchange-correlation energy with respect to the electronic charge density and magnetization, respectively. The electronic bandstructure of the disordered alloy is then computed via a self-consistent Korringa–Kohn–Rostoker approach [26,27] for the coherent potential approximation [28,29,30] employing the potentials found above.

In this work, we describe electron correlations in disordered magnetic crystals based on a self-consistent Green’s function method for the multiband Hamiltonian; Green’s functions are found via a diagrammatic approach. Electron–electron interactions, electron–phonon interactions, and disorder effects are all incorporated into the theory. We begin by determining the wave functions for the noninteracting atoms via the Kohn–Sham equation. The effective one-electron potential of the many-atom structure is approximated as a sum of spherical Kohn–Sham potentials of neutral noninteracting atoms. The potential of the neutral atoms is found by the meta-generalized gradient approximation (MGGA) [8,10]. The wavefunctions and atomic potentials are found self-consistently, by taking into account the redistribution of the electron density as a result of the atomic interactions. This also includes the long-range Coulomb interaction of the electrons on different sites of the crystal lattice. Electron scattering processes from the ionic core potentials of the different atomic sites and from the oscillations of the crystal lattice (phonons) are also included. First we calculate Green’s functions for imaginary time [31]. They are then related to the real-time Green’s functions via an analytic continuation that employs the standard spectral representation [32].

This calculation of real-time Green’s functions of the disordered crystal is based on a diagrammatic technique, analogous to the technique used for a homogeneous system [32]. The set of equations of real-time Green’s functions, along with expressions for the free energy and the electrical conductivity of disordered crystals, is based on the work presented in [31]. These methods are employed to obtain our final results. In the next two sections, we sketch the formalism to establish our notation and summarize the methods used.

## 2. Hamiltonian of an Electron–Phonon System for a Disordered Crystal

The many-body Hamiltonian of a disordered crystal consists of a single-particle Hamiltonian of the electrons in the external potential of the (disordered) ionic cores, the potential energy of the electron–electron interaction, the quadratic Hamiltonian for the phonons, the contribution from the electron–phonon interaction, and the anharmonic phonon potential terms.

We represent the Hamiltonian in the basis of free neutral atoms. In the Wannier representation, the system Hamiltonian is [31]

(1) $$H=H_{0}+H_{\mathrm{int}}$$

where the zeroth-order Hamiltonian

(2) $$H_{0}=H_{e}^{\left(0\right)}+H_{ph}^{\left(0\right)}$$

consists of the single-particle Hamiltonian of the electrons in the field of the ionic cores

(3) $$H_{e}^{\left(0\right)}=\sum_{\begin{array}{c} ni\gamma \\ n^{\prime }i^{\prime }\gamma^{\prime } \end{array}}h_{ni\gamma ,n^{\prime }i^{\prime }\gamma^{\prime }}^{\left(0\right)}a_{ni\gamma }^{+}a_{n^{\prime }i^{\prime }\gamma^{\prime }}$$

and the harmonic phonon Hamiltonian for the motion of the ion cores

(4) $$H_{ph}^{\left(0\right)}=\sum_{ni\alpha }\frac{P_{ni\alpha }^{2}}{2M_{i}}+\frac{1}{2}\sum_{\begin{array}{c} ni\alpha \\ n^{\prime }i^{\prime }\alpha^{\prime } \end{array}}\Phi_{ni\alpha ,n^{\prime }i^{\prime }\alpha^{\prime }}^{\left(0\right)}u_{ni\alpha }u_{n^{\prime }i^{\prime }\alpha^{\prime }}.$$

Here, the ion cores are located on a periodic lattice (i.e., the unperturbed system is periodically ordered and has no disorder). The symbol *n* denotes the unit cell, *i* denotes the *i*th basis vector in the *n*th unit cell, and γ denotes all of the other quantum numbers for the orbital, including spin. Disorder will enter for the species of ion at a particular lattice site, which need not be periodic via a perturbed Hamiltonian term (see below). The symbol $h^{\left(0\right)}$ denotes the “hopping integral” that connects the respective orbitals. For the phonon Hamiltonian, *n* and *i* are the same as before, namely the unit cell and basis site within the unit cell, while α is a spatial direction (x,y, or z). *P* is the ionic momentum, *M* is the ionic mass, *u* is the deviation of the ion from the equilibrium position of the lattice site, and $\Phi^{\left(0\right)}$ is the corresponding spring-constant matrix.

The interaction Hamiltonian in Equation (1) is the perturbation of the system due to all of the effects we will be including. It is composed of six pieces:

(5) $$H_{\mathrm{int}}=\delta \Phi +H_{ei}+H_{eph}+H_{ee}+H_{phi}+H_{phph}.$$

δΦ is the modification of the ion-core—ion-core Coulomb interaction due to the disordered ions added to the system; it is the difference between the original ion–ion repulsion Hamiltonian and the new one. The single-particle electronic Hamiltonian is modified by the change in the ion core and the extra term:

(6) $$H_{ei}=\sum_{\begin{array}{c} ni\gamma \\ n^{\prime }i^{\prime }\gamma^{\prime } \end{array}}w_{ni\gamma ,n^{\prime }i^{\prime }\gamma^{\prime }}a_{ni\gamma }^{+}a_{n^{\prime }i^{\prime }\gamma^{\prime }},$$

which is the difference between the new hopping Hamiltonian and the original periodic one. The electron–phonon interaction is given by

(7) $$H_{eph}=\sum_{\begin{array}{c} ni\gamma \\ n^{\prime }i^{\prime }\gamma^{\prime } \end{array}}v_{ni\gamma ,n^{\prime }i^{\prime }\gamma^{\prime }}^{\prime }a_{ni\gamma }^{+}a_{n^{\prime }i^{\prime }\gamma^{\prime }}.$$

This is described in more detail below. The screened Coulomb interaction between electrons is given by the different multiband interaction terms, including density–density interactions and exchange interactions

(8) $$H_{ee}=\frac{1}{2}\;\sum_{\begin{array}{c} ni\gamma \\ n{}^{\prime }i{}^{\prime }\gamma {}^{\prime }\\ n{}^{\prime \prime }i{}^{\prime \prime }\gamma {}^{\prime \prime }\\ n{}^{\prime \prime \prime }i{}^{\prime \prime \prime }\gamma {}^{\prime \prime \prime } \end{array}}\;v_{n{}^{\prime \prime }i{}^{\prime \prime }\gamma {}^{\prime \prime },n{}^{\prime \prime \prime }i{}^{\prime \prime \prime }\gamma {}^{\prime \prime \prime }}^{\left(2\right)ni\gamma ,n{}^{\prime }i{}^{\prime }\gamma {}^{\prime }}a_{ni\gamma }^{+}a_{n{}^{\prime }i{}^{\prime }\gamma {}^{\prime }}^{+}a_{n{}^{\prime \prime }i{}^{\prime \prime }\gamma {}^{\prime \prime }}a_{n{}^{\prime \prime \prime }i{}^{\prime \prime \prime }\gamma {}^{\prime \prime \prime }}.$$

The modification of the interaction of the phonons with the impurity ion cores is given by

(9) $$\begin{array}{ccc} H_{phi} & = & \frac{1}{2}\sum_{\begin{array}{c} ni\alpha \\ n^{\prime }i^{\prime }\alpha^{\prime } \end{array}}\Delta M_{ni\alpha ,n^{\prime }i^{\prime }\alpha^{\prime }}^{-1}P_{ni\alpha }P_{n^{\prime }i^{\prime }\alpha^{\prime }}\\ & + & \frac{1}{2}\sum_{\begin{array}{c} ni\alpha \\ n^{\prime }i^{\prime }\alpha^{\prime } \end{array}}\Delta \Phi_{ni\alpha ,n^{\prime }i^{\prime }\alpha^{\prime }}u_{ni\alpha }u_{n^{\prime }i^{\prime }\alpha^{\prime }} \end{array}$$

where

(10) $$\Delta M_{ni\alpha ,n^{\prime }i^{\prime }\alpha^{\prime }}^{-1}=\left(\frac{1}{M_{ni^{\prime }}}-\frac{1}{M_{i}}\right)\delta_{nn^{\prime }}\delta_{ii^{\prime }}\delta_{\alpha \alpha^{\prime }}$$

(11) $$\Delta \Phi_{ni\alpha ,n^{\prime }i^{\prime }\alpha^{\prime }}=\Phi_{ni\alpha ,n^{\prime }i^{\prime }\alpha^{\prime }}-\Phi_{ni\alpha ,n^{\prime }i^{\prime }\alpha^{\prime }}^{\left(0\right)}$$

and $M_{ni}$ and $M_{i}$ are the masses of the atoms at site (ni) for disordered and ordered alloys, respectively.

We also include the cubic anharmonic potential terms for the phonons (under the assumption that they remain small and can be treated perturbatively) via

(12) $$\begin{array}{c} H_{phph}\;=\frac{1}{3!}\;\sum_{\begin{array}{c} ni\alpha \\ n{}^{\prime }i{}^{\prime }\alpha {}^{\prime }\\ n{}^{\prime \prime }i{}^{\prime \prime }\alpha {}^{\prime \prime } \end{array}}\;\Phi_{ni\alpha ,n{}^{\prime }i{}^{\prime }\alpha {}^{\prime },n{}^{\prime \prime }i{}^{\prime \prime }\alpha {}^{\prime \prime }}^{\left(0\right)}u_{ni\alpha }u_{n{}^{\prime }i{}^{\prime }\alpha {}^{\prime }}u_{n{}^{\prime \prime }i{}^{\prime \prime }\alpha {}^{\prime \prime }}. \end{array}$$

The strategy for determining the matrix elements of the Hamiltonian is to employ the conventional density functional theory within the generalized gradient approximation to solve the Kohn–Sham equations in the presence of a Hartree plus exchange-correlation potential. We then employ those wavefunctions as the basis for expanding the full Hamiltonian, where we include explicitly the Coulomb repulsion of the electrons, which avoids the double-counting problem usually associated with trying to add correlations on top of density functional theory (which includes some correlation effects at the mean-field level).

More concretely, the strategy is as follows: We first determine the electronic wavefunctions from the standard density functional theory approach. The operators $a_{ni\gamma }^{+}$, $a_{ni\gamma }$ create and destroy electrons in the state described by Vane’s function $\phi_{ni\gamma }\left(\xi \right)=\langle \xi |ni\gamma \rangle$, where ξ=(r,σ) are the spatial and *z*-component of spin coordinates of the wavefunction [33]. These wavefunctions (of an electron in the field of a free neutral atom species λ located at site (ni) are obtained from the Kohn–Sham equation in density functional theory [10]:

(13) $$\begin{array}{l} \left[-\frac{\hbar^{2}}{2m}\nabla^{2}+V_{ext}^{\lambda }\left(r\right)+V_{H}^{\lambda i}\left(r\right)+V_{XC,\sigma }^{\lambda i}\left(r\right)\right]\\ \times \varphi_{i\gamma }\left(r\right)=\varepsilon_{i\tilde{\varepsilon }l\sigma }^{\lambda }\varphi_{i\gamma }\left(r\right) \end{array}$$

where γ is a superindex which incorporates the quantum numbers for the principle energy eigenvalue $\tilde{\varepsilon }$, the standard angular momentum quantum numbers *l* and *m*, and the *z*-component of spin σ. To reduce the length of Equation (13), the relative coordinate $\left(r-r_{ni}\right)$ is denoted by the simplification (r).

In Equation (13), $V_{ext}^{\lambda }\left(r\right)$ is the Hartree potential energy for an electron in the atomic core Coulomb field of type λ at basis site *i*, which is given by the simple expression

(14) $$V_{H}^{\lambda i}\left(r\right)=\int dv^{\prime }\frac{e^{2}}{\left|r-r^{\prime }\right|}n_{\lambda i}\left(r^{\prime }\right).$$

In Equation (14), the electron density is summed over both spin components:

(15) $$n_{\lambda i}\left(r\right)=n_{\lambda i\sigma }\left(r\right)+n_{\lambda i-\sigma }\left(r\right).$$

The electron density for each component of spin is given by

(16) $$n_{\lambda i\sigma }\left(r\right)=\sum_{\tilde{\varepsilon }lm}Z_{ni\gamma }^{\lambda }\varphi_{ni\gamma }^{*}\left(r\right)\varphi_{ni\gamma }\left(r\right)$$

where $Z_{ni\gamma }^{\lambda }$ is the occupation number electrons in the state denoted by γ (of type λ at basis site *i* and with spin component σ).

The exchange-correlation potential is more complicated. In the MGGA [8,10], it is represented by

(17) $$\begin{array}{ccc} V_{XC,\sigma }^{MGGA}\left(r\right)\psi_{\gamma \sigma }\left(r\right) & = & V_{XC,\sigma }^{GGA}\left(r\right)\psi_{\gamma \sigma }\left(r\right)\\ & - & \frac{1}{2}\nabla \left\{\mu_{XC,\sigma }\left(r\right)\nabla \right\}\psi_{\gamma \sigma }\left(r\right) \end{array}$$

where

(18) $$V_{XC,\sigma }^{GGA}\left(r\right)=\left[\frac{\partial e_{XC}^{MGGA}}{\partial n_{\sigma }}-\nabla \left(\frac{\partial e_{XC}^{MGGA}}{\partial \nabla n_{\sigma }}\right)\right]$$

is the GGA contribution, and $\mu_{XC,\sigma }\left(r\right)=\frac{\partial e_{XC}^{MGGA}}{\partial \tau_{\sigma }}$ is one of the correction terms. Here, $e_{XC}^{MGGA}\left(2n_{\sigma }\right)/2$ is the exchange-correlational energy density, and $\tau_{\sigma }=\sum_{\delta }{\left|\nabla \psi_{\delta \sigma }\right|}^{2}/2$ is the kinetic energy density for each spin component.

The energy eigenstate wavefunctions corresponding to the periodic Hamiltonian in Equation (1) are denoted by $\varphi_{ni\gamma }\left(r\right)$. They are found by solving the Kohn–Sham equations in Equation (13) and are expressed in a factorized form for the radial and angular components via

(19) $$\varphi_{ni\gamma }\left(r\right)=R_{i\tilde{\varepsilon }l\sigma }^{\lambda }\left(\left|r-r_{ni}\right|\right)Y_{lm}\left(\theta ,\varphi \right)$$

where $R_{i\tilde{\varepsilon }l\sigma }^{\lambda }\left(\left|r-r_{ni}\right|\right)$ is the radial component and we employ the spherical functions $Y_{lm}\left(\theta ,\varphi \right)$ for the angular component.

The potential-energy operator of an electron in the field of the different ionic cores is given by

(20) $$V\left(r\right)=\sum_{ni}v^{ni}\left(r-{r^{\prime }}_{ni}\right),\;{r^{\prime }}_{ni}=r_{ni}+u_{ni}^{s}+u_{ni}$$

where **r** is the electron position vector, $r_{ni}=r_{n}+\rho_{i}$ is the position vector for the ion core at site (ni) in equilibrium, and $u_{ni}^{s}$ ionic core’s static displacement from equilibrium due to phonons, and $u_{ni}$ is the dynamic ion core displacement due to phonons. The total potentials of the ionic core $v^{ni}\left(r-r_{ni}\right)$ are found from Equation (20) and require a summation over all electronic states.

The matrix element of the electron-ion interaction Hamiltonian in Equation (6) is given by

(21) $$w_{ni\gamma ,n{}^{\prime }i{}^{\prime }\gamma {}^{\prime }}=\sum_{n{}^{\prime \prime }i{}^{\prime \prime }}w_{ni\gamma ,n{}^{\prime }i{}^{\prime }\gamma {}^{\prime }}^{n{}^{\prime \prime }i{}^{\prime \prime }}$$

where

(22) $$w_{ni\gamma ,n{}^{\prime }i{}^{\prime }\gamma {}^{\prime }}^{n{}^{\prime \prime }i{}^{\prime \prime }}=\sum_{\lambda }c_{n{}^{\prime \prime }i{}^{\prime \prime }}^{\lambda }w_{ni\gamma ,n{}^{\prime }i{}^{\prime }\gamma {}^{\prime }}^{\lambda n{}^{\prime \prime }i{}^{\prime \prime }}$$

(23) $$w_{ni\gamma ,n{}^{\prime }i{}^{\prime }\gamma {}^{\prime }}^{\lambda n{}^{\prime \prime }i{}^{\prime \prime }}=v_{ni\gamma ,n{}^{\prime }i{}^{\prime }\gamma {}^{\prime }}^{\lambda n{}^{\prime \prime }i{}^{\prime \prime }}+\Delta v_{ni\gamma ,n{}^{\prime }i{}^{\prime }\gamma {}^{\prime }}^{\lambda n{}^{\prime \prime }i{}^{\prime \prime }}-v_{ni\gamma ,n{}^{\prime }i{}^{\prime }\gamma {}^{\prime }}^{\lambda i{}^{\prime \prime }n{}^{\prime \prime }i{}^{\prime \prime }}$$

with $\lambda_{i{}^{\prime \prime }}$ the type of ion at $\left(n{}^{\prime \prime }i{}^{\prime \prime }\right)$. Here, $c_{ni}^{\lambda }$ are random numbers, taking the values 1 or 0, depending on whether the atom of type λ is at site (ni) or not. The symbol ν is a matrix element of the potential of the ionic core $v^{ni}\left(r-r_{ni}\right)$. The symbol Δv will be defined next.

The expression for the electron–phonon interaction in Equation (7) is found through derivatives of the potential energy of the electrons in the ion core field due to a displacement of the atom by the vector $u_{ni}$. In Equation (7), the value of $v_{ni\gamma ,n^{\prime }i^{\prime }\gamma^{\prime }}^{\prime }$ is given by

(24) $$v_{ni\gamma ,n{}^{\prime }i{}^{\prime }\gamma {}^{\prime }}^{{}^{\prime }}=\sum_{n{}^{\prime \prime }i{}^{\prime \prime }\alpha }{v{}^{\prime }}_{ni\gamma ,n{}^{\prime }i{}^{\prime }\gamma {}^{\prime }}^{n{}^{\prime \prime }i{}^{\prime \prime }\alpha }u_{n{}^{\prime \prime }i{}^{\prime \prime }\alpha }$$

where

(25) $${v^{\prime }}_{ni\gamma ,n{}^{\prime }i{}^{\prime }\gamma {}^{\prime }}^{n{}^{\prime \prime }i{}^{\prime \prime }\alpha }=\sum_{\lambda }c_{n{}^{\prime \prime }i{}^{\prime \prime }}^{\lambda }{v{}^{\prime }}_{ni\gamma ,n{}^{\prime }i{}^{\prime }\gamma {}^{\prime }}^{\lambda n{}^{\prime \prime }i{}^{\prime \prime }\alpha }$$

with ${v{}^{\prime }}_{ni\gamma ,n{}^{\prime }i{}^{\prime }\gamma {}^{\prime }}^{\lambda n{}^{\prime \prime }i{}^{\prime \prime }\alpha }$ the matrix elements of the following operator

(26) $$-e_{n{}^{\prime \prime }i{}^{\prime \prime }\alpha }\frac{d}{d\left|{r-r}_{n{}^{\prime \prime }i{}^{\prime \prime }}\right|}v^{\lambda }\left(\left|{r-r}_{n{}^{\prime \prime }i{}^{\prime \prime }}\right|\right)$$

where

(27) $$e_{n{}^{\prime \prime }i{}^{\prime \prime }}=\frac{{r-r}_{n{}^{\prime \prime }i{}^{\prime \prime }}}{\left|{r-r}_{n{}^{\prime \prime }i{}^{\prime \prime }}\right|}.$$

The term $\Delta v_{ni\gamma ,n{}^{\prime }i{}^{\prime }\gamma {}^{\prime }}^{\lambda n{}^{\prime \prime }i{}^{\prime \prime }}$ in Equation (21) describes electron scattering on the static displacement of the atoms and is defined by the equation

(28) $$\Delta v_{ni\gamma ,n{}^{\prime }i{}^{\prime }\gamma {}^{\prime }}^{\lambda n{}^{\prime \prime }i{}^{\prime \prime }}=\sum_{\alpha }{v{}^{\prime }}_{ni\gamma ,n{}^{\prime }i{}^{\prime }\gamma {}^{\prime }}^{\lambda n{}^{\prime \prime }i{}^{\prime \prime }\alpha }u_{n{}^{\prime \prime }i{}^{\prime \prime }\alpha }^{s}.$$

The matrix of the force constants arising from the direct Coulomb interaction of the ionic cores has the form

(29) $$\begin{array}{ccc} \Phi_{ni\alpha ,n^{\prime }i^{\prime }\alpha^{\prime }} & = & -\frac{Z_{ni}Z_{n^{\prime }i^{\prime }}e^{2}}{4\pi \varepsilon_{0}{\left|r_{n}+\rho_{i}-r_{n^{\prime }}-\rho_{i^{\prime }}\right|}^{5}}\\ & \times & [3\left(r_{n\alpha }+\rho_{i\alpha }-r_{n^{\prime }\alpha }-\rho_{i^{\prime }\alpha }\right)\\ & \times & \left(r_{n\alpha^{\prime }}+\rho_{i\alpha^{\prime }}-r_{n^{\prime }\alpha^{\prime }}-\rho_{i^{\prime }\alpha^{\prime }}\right)\\ & - & {\left|r_{n}+\rho_{i}-r_{n^{\prime }}-\rho_{i^{\prime }}\right|}^{2}\delta_{\alpha \alpha^{\prime }}]\\ & & \left(ni\right)\neq \left(n^{\prime }i^{\prime }\right) \end{array}$$

where $Z_{ni}$ is the valence of the ion cores at (ni). This matrix satisfies the following constraint:

(30) $$\sum_{n^{\prime }i^{\prime }}\Phi_{ni\alpha ,n^{\prime }i^{\prime }\alpha^{\prime }}=0.$$

The force constants with the (0) superscript are defined in the same fashion, but correspond to the force constants of the initial periodic system with no disorder.

The matrix elements $v_{n{}^{\prime \prime }i{}^{\prime \prime }\gamma {}^{\prime \prime },n{}^{\prime \prime \prime }i{}^{\prime \prime \prime }\gamma {}^{\prime \prime \prime }}^{\left(2\right)ni\gamma ,n{}^{\prime }i{}^{\prime }\gamma {}^{\prime }}$ in Equation (8) are calculated by integrating over the corresponding angular variables. Integrals of the product of three spherical functions (a so-called Gaunt integral) are found by using Clebsch–Gordan coefficients [34,35]. This yields

(31) $$\begin{array}{l} v_{\tilde{\varepsilon }{}^{\prime \prime }l{}^{\prime \prime }m{}^{\prime \prime },\tilde{\varepsilon }{}^{\prime \prime \prime }l{}^{\prime \prime \prime }m{}^{\prime \prime \prime }}^{\left(2\right)\tilde{\varepsilon }lm,\tilde{\varepsilon }{}^{\prime }l{}^{\prime }m{}^{\prime }}=e^{2}\sum_{\begin{array}{c} \left|l-l{}^{\prime \prime \prime }\right|⩽l_{1}⩽l+l{}^{\prime \prime \prime }\\ \left|l{}^{\prime }-l{}^{\prime \prime }\right|⩽l_{1}⩽l{}^{\prime }+l{}^{\prime \prime }\\ l+l{}^{\prime \prime \prime }+l_{1}=2k,k\in N\\ l{}^{\prime }+l{}^{\prime \prime }+l_{1}=2k_{1},k_{1}\in N \end{array}}\;\frac{1}{2l_{1}+1}\\ \times {\left[\frac{\left(2l_{1}+1\right)\left(2l{}^{\prime \prime \prime }+1\right)\left(2l_{1}+1\right)\left(2l{}^{\prime \prime }+1\right)}{\left(2l+1\right)\left(2l{}^{\prime }+1\right)}\right]}^{1/2}\\ \times c\left(l_{1}l{}^{\prime \prime \prime }l;0,0\right)c\left(l_{1}l{}^{\prime \prime \prime }l;m{}^{\prime \prime \prime }-m,m{}^{\prime \prime \prime }\right)\\ \times c\left(l_{1}l{}^{\prime \prime }l{}^{\prime };0,0\right)c\left(l_{1}l{}^{\prime \prime }l{}^{\prime };m{}^{\prime }-m{}^{\prime \prime },m{}^{\prime \prime }\right)\\ \times \left[\int_{0}^{\infty }dr_{1}r_{1}^{2}\right.R_{\tilde{\varepsilon }l}\left(r_{1}\right)R_{\tilde{\varepsilon }{}^{\prime \prime \prime }l{}^{\prime \prime \prime }}\left(r_{1}\right)\\ \times \int_{0}^{r_{1}}dr_{2}r_{2}^{2}R_{\tilde{\varepsilon }{}^{\prime }l{}^{\prime }}\left(r_{2}\right)R_{\tilde{\varepsilon }{}^{\prime \prime }l{}^{\prime \prime }}\left(r_{2}\right)\frac{r_{2}^{l_{1}}}{r_{1}^{l_{1}+1}}\\ +\int_{0}^{\infty }dr_{2}r_{2}^{2}R_{\tilde{\varepsilon }{}^{\prime }l{}^{\prime }}\left(r_{2}\right)R_{\tilde{\varepsilon }{}^{\prime \prime }l{}^{\prime \prime }}\left(r_{2}\right)\\ \times \left.\int_{0}^{r_{2}}dr_{1}r_{1}^{2}R_{\tilde{\varepsilon }l}\left(r_{1}\right)R_{\tilde{\varepsilon }{}^{\prime \prime \prime }l{}^{\prime \prime \prime }}\left(r_{1}\right)\frac{r_{1}^{l_{1}}}{r_{2}^{l_{1}+1}}\right] \end{array}$$

where *l* and *m* are the standard angular momentum quantum numbers, $c(l{}^{\prime \prime }l{}^{\prime }l;m{}^{\prime \prime },m{}^{\prime })$ are the standard Clebsch–Gordan coefficients [34], $R_{\tilde{\varepsilon }\;l}\left(r\right)$ is the radial part of wave function, and $\tilde{\varepsilon }$ is the principle quantum number. There is a further simplification that we invoke when we treat the system with Gaussian orbitals. Thus, the matrix elements in the real wave function basis $v_{n{}^{\prime \prime }i{}^{\prime \prime }\gamma {}^{\prime \prime },n{}^{\prime \prime \prime }i{}^{\prime \prime \prime }\gamma {}^{\prime \prime \prime }}^{\left(2\right)ni\gamma ,n{}^{\prime }i{}^{\prime }\gamma {}^{\prime }}$ for different sites (ni) are approximately represented in a form similar to that in Equation (19). When the radial part is a Gaussian orbital, as is done in the method of molecular orbitals via linear combinations of the atomic orbitals${}^{12}$, multicenter integrals $v_{n{}^{\prime \prime }i{}^{\prime \prime }\gamma {}^{\prime \prime },n{}^{\prime \prime \prime }i{}^{\prime \prime \prime }\gamma {}^{\prime \prime \prime }}^{\left(2\right)ni\gamma ,n{}^{\prime }i{}^{\prime }\gamma {}^{\prime }}$ have the form of single-center integrals, because the product of two Gaussian orbitals that are localized at different centers can be reduced to the product of orbitals that are localized about a common center.

## 3. Green’s Functions of Electrons and Phonons

We employ a Green’s function-based formalism to perform the calculations. Ultimately, we need the real-time retarded $G_{r}^{AB}\left(t,t{}^{\prime }\right)$ and advanced $G_{a}^{AB}\left(t,t{}^{\prime }\right)$ Green’s functions, which are each defined as follows [35]:

(32) $$\begin{array}{ccc} G_{r}^{AB}\left(t,t{}^{\prime }\right) & = & -\frac{i}{\hbar }\theta \left(t-t{}^{\prime }\right)\langle \left[\tilde{A}\left(t\right),\tilde{B}\left(t{}^{\prime }\right)\right]\rangle ,\\ G_{a}^{AB}\left(t,t{}^{\prime }\right) & = & \frac{i}{\hbar }\theta \left(t{}^{\prime }-t\right)\langle \left[\tilde{A}\left(t\right),\tilde{B}\left(t{}^{\prime }\right)\right]\rangle . \end{array}$$

Here, the operators are expressed in the Heisenberg representation

(33) $$\tilde{A}\left(t\right)=e^{iHt/\hbar }Ae^{-iHt/\hbar }$$

where *ℏ* is Planck’s constant, $H=H-\mu_{e}N_{e}$, $\mu_{e}$ is the chemical potential of the electronic subsystem, and $N_{e}$ is the electron number operator given by

(34) $$N_{e}=\sum_{ni\gamma }a_{ni\gamma }^{+}a_{ni\gamma }.$$

In addition, the commutator or anticommutator is defined via

(35) [A,B]=AB∓BA

where the commutator is used for Bose operators (–), and the anticommutator is used for Fermi operators (+). The symbol θ(t) is Heaviside’s unit step function. The angle brackets 〈…〉 denote the thermal averaging with respect to the density matrix ρ

(36) $$\langle A\rangle =\mathrm{Tr}\left(\rho A\right),\;\rho =e^{(\Omega -H)/\Theta }$$

where Ω is the thermodynamic potential of the system given by exp(Ω/Θ)=Trexp(−H/Θ) and $\Theta =k_{b}T$, with $k_{b}$ Boltzmann’s constant and *T* the temperature. Note that, even though the real-time Green’s functions appear to depend on two different times, because of the time-translational invariance for equilibrium systems, they actually depend only on the time difference $t-t{}^{\prime }$.

Our procedure for calculating the real-time Green’s functions follows the standard one—we first determine the thermal Green’s functions (defined below) and then analytically continue using them as real-time functions using the conventional spectral relations.

The thermal Green’s function are defined by

(37) $$G^{AB}\left(\tau ,\tau {}^{\prime }\right)=-\langle T_{\tau }\tilde{A}\left(\tau \right)\tilde{B}\left(\tau {}^{\prime }\right)\rangle$$

where the imaginary-time operator $\tilde{A}\left(\tau \right)$ is derived from the real-time Heisenberg representation and the substitution t=−iℏτ. Hence,

(38) $$\tilde{A}\left(\tau \right)=e^{H\tau }Ae^{-H\tau }.$$

In addition, the time-ordering operator satisfies

(39) $$\begin{array}{ccc} T_{\tau }\tilde{A}\left(\tau \right)\tilde{B}\left(\tau {}^{\prime }\right) & = & \theta \left(\tau -\tau {}^{\prime }\right)\tilde{A}\left(\tau \right)\tilde{B}\left(\tau {}^{\prime }\right)\\ & \pm & \theta \left(\tau {}^{\prime }-\tau \right)\tilde{B}\left(\tau {}^{\prime }\right)\tilde{A}\left(\tau \right) \end{array}$$

where the plus sign is used for Bose operators and the minus sign for Fermi operators.

We next go to the interaction representation by introducing the operator

(40) $$\sigma \left(\tau \right)=e^{H_{0}\tau }e^{-H\tau },$$

with $H=H_{0}+H_{\mathrm{int}}$ and $H_{0}=H_{0}-\mu_{e}N_{e}$.

Differentiating the expression for σ(τ) in Equation (40) with respect to τ and then integrating from 0 with the boundary condition σ(0)=1, we obtain

(41) $$\sigma \left(\tau \right)=T_{\tau }\exp\left[-\int_{0}^{\tau }H_{\mathrm{int}}\left(\tau {}^{\prime }\right)d\tau {}^{\prime }\right]$$

where $H_{\mathrm{int}}\left(\tau \right)=e^{H_{0}\tau }H_{\mathrm{int}}e^{-H_{0}\tau }$. Employing this result yields

(42) $$\tilde{A}\left(\tau \right)=\sigma^{-1}\left(\tau \right)A\left(\tau \right)\sigma \left(\tau \right)$$

with A(τ) in the Heisenberg representation with respect to the noninteracting Hamiltonian. Substituting these results into the definition of the thermal Green’s function creates the alternate interaction-representation form for the Green’s function, given by

(43) $$G^{AB}\left(\tau ,\tau {}^{\prime }\right)=-\frac{{\langle T_{\tau }A\left(\tau \right)B\left(\tau {}^{\prime }\right)\sigma \left(1/\Theta \right)\rangle }_{0}}{{\langle \sigma \left(1/\Theta \right)\rangle }_{0}}$$

where all time dependence is with respect to the noninteracting Hamiltonian and the trace over all states is with respect to the noninteracting states

(44) $${\langle A\rangle }_{0}=\mathrm{Tr}\left(\rho_{0}A\right),\;\rho_{0}=e^{(\Omega_{0}-H_{0})/\Theta }.$$

This last result forms the starting point for the perturbative expansion employed here.

The diagrammatic method is generated by expanding σ(τ) in a power series in terms of $H_{\mathrm{int}}\left(\tau \right)$ and then using Wick’s theorem to evaluate the resulting operator averages (since the noninteracting Hamiltonian is quadratic [31]). This technique then generalizes the approach used for the homogeneous system [31]. The denominator in Equation (43) cancels all disconnected diagrams in the expansion, as usual. Therefore, the thermal Green’s function are expanded in terms of connected diagrams. Using the standard relations between the spectral representations of thermal and real-time Green’s functions [31], we obtain the following Dyson equation for the electronic Green’s function in the real frequency domain (hereinafter the dependence on *r* is suppressed) [31]:

(45) $$\begin{array}{ccc} G^{aa^{+}}\left(\varepsilon \right) & = & G_{0}^{aa^{+}}\left(\varepsilon \right)+G_{0}^{aa^{+}}\left(\varepsilon \right)\\ & \times & \left(w+\Sigma_{eph}\left(\varepsilon \right)+\Sigma_{ee}\left(\varepsilon \right)\right)G^{aa^{+}}\left(\varepsilon \right)\\ G^{uu}\left(\varepsilon \right) & = & G_{0}^{uu}\left(\varepsilon \right)+G_{0}^{uu}\left(\varepsilon \right)\left(\Delta \Phi +\Sigma_{phe}\left(\varepsilon \right)+\Sigma_{phph}\left(\varepsilon \right)\right)\\ & \times & G^{uu}\left(\varepsilon \right)+G_{0}^{uP}\left(\varepsilon \right)\Delta M^{-1}G^{Pu}\left(\varepsilon \right)\\ G^{PP}\left(\varepsilon \right) & = & G_{0}^{PP}\left(\varepsilon \right)+G_{0}^{PP}\left(\varepsilon \right)\Delta M^{-1}G^{PP}\left(\varepsilon \right)+G_{0}^{Pu}\left(\varepsilon \right)\\ & \times & \left(\Delta \Phi +\Sigma_{phe}\left(\varepsilon \right)+\Sigma_{phph}\left(\varepsilon \right)\right)G^{uP}\left(\varepsilon \right)\\ G^{uP}\left(\varepsilon \right) & = & G_{0}^{uP}\left(\varepsilon \right)+G_{0}^{uP}\left(\varepsilon \right)\Delta M^{-1}G^{PP}\left(\varepsilon \right)+G_{0}^{uu}\left(\varepsilon \right)\\ & \times & \left(\Delta \Phi +\Sigma_{phe}\left(\varepsilon \right)+\Sigma_{phph}\left(\varepsilon \right)\right)G^{uP}\left(\varepsilon \right)\\ G^{Pu}\left(\varepsilon \right) & = & G_{0}^{Pu}\left(\varepsilon \right)+G_{0}^{Pu}\left(\varepsilon \right)\left(\Delta \Phi +\Sigma_{phe}\left(\varepsilon \right)+\Sigma_{phph}\left(\varepsilon \right)\right)\\ & \times & G^{uu}\left(\varepsilon \right)+G_{0}^{PP}\left(\varepsilon \right)\Delta M^{-1}G^{Pu}\left(\varepsilon \right) \end{array}$$

where ε=ℏω. Here $G^{aa^{+}}\left(\varepsilon \right)$, $G^{uu}\left(\varepsilon \right)$, $G^{PP}\left(\varepsilon \right)$, $G^{uP}\left(\varepsilon \right)$, and $G^{Pu}\left(\varepsilon \right)$ are the real-frequency representations of the single-particle Green’s function of the electrons, the coordinate–coordinate, momentum–momentum, coordinate–momentum, and momentum–coordinate Green’s functions of the phonons, respectively; $\Sigma_{eph}\left(\varepsilon \right)$, $\Sigma_{phe}\left(\varepsilon \right)$, $\Sigma_{ee}\left(\varepsilon \right)$, and $\Sigma_{phph}\left(\varepsilon \right)$ are the corresponding self-energies (mass operators) for the electron–phonon, phonon–electron, electron–electron, and phonon–phonon interactions.

The real-time and real-frequency Green’s functions are related by standard Fourier transform relations given by

(46) $$G_{r,a}^{AB}\left(t\right)=\frac{1}{2\pi }\int_{-\infty }^{\infty }G_{r,a}^{AB}\left(\omega \right)e^{-i\omega t}d\omega$$

and

(47) $$G_{r,a}^{AB}\left(\omega \right)=\int_{-\infty }^{\infty }G_{r,a}^{AB}\left(t\right)e^{i\omega t}dt.$$

The thermal Green’s functions are periodic (bosons) or antiperiodic (fermions) on the interval −1/Θ⩽τ<1/Θ, and hence have a Fourier series representation in terms of their Matsubara frequencies, as follows:

(48) $$G^{AB}\left(\tau \right)=\Theta \sum_{\omega_{n}}G^{AB}\left(\omega_{n}\right)\;e^{-i\omega_{n}\tau }$$

and

(49) $$G^{AB}\left(\omega_{n}\right)=\frac{1}{2}\int_{-1/\Theta }^{1/\Theta }G^{AB}\left(\tau \right)\;e^{i\omega_{n}\tau }d\tau$$

where the Matsubara frequencies satisfy

(50) $$\begin{array}{c} \omega_{n}=\left\{\begin{array}{cc} 2n\pi \;\Theta & \mathrm{for}\;\mathrm{Bose}\;\mathrm{particles}\\ (2n+1)\pi \;\Theta & \mathrm{for}\;\mathrm{Fermi}\;\mathrm{particles} \end{array}\right.\\ n=0,\pm 1,\pm 2,\ldots \;. \end{array}$$

Note that the thermal Green’s functions are equal to the retarded Green’s functions evaluated at the Matsubara frequencies for positive Matusbara frequencies and are equal to the advanced Green’s functions evaluated at the Matsubara frequencies for negative Matsubara frequencies.

The electronic Green’s functions are infinite matrices with indices given by the lattice site *n*, the basis site *i*, and the other quantum numbers γ. Similarly, the phonon Green’s functions are also infinite matrices with the same lattice and basis site dependence plus a dependence on the spatial coordinate direction α. This produces some simple equations for the noninteracting Green’s functions, namely [31]

(51) $$G_{0}^{aa^{+}}\left(\varepsilon \right)={\left[\varepsilon -H_{0}^{\left(1\right)}\right]}^{-1}$$

with

(52) $$H_{0}^{\left(1\right)}=∥h_{ni\gamma ,n^{\prime }i^{\prime }\gamma^{\prime }}^{\left(0\right)}∥$$

(53) $$G_{0}^{uu}\left(\varepsilon \right)={\left[\omega^{2}M^{\left(0\right)}-\Phi^{\left(0\right)}\right]}^{-1}$$

(54) $$\Phi^{\left(0\right)}=∥\Phi_{ni\alpha ,n^{\prime }i^{\prime }\alpha^{\prime }}^{\left(0\right)}∥$$

and

(55) $$M^{\left(0\right)}=∥M_{i}\delta_{nn^{\prime }}\delta_{ii^{\prime }}\delta_{\alpha \alpha^{\prime }}∥.$$

Here, the double lines denote a matrix.

When the perturbations are small, given by

(56) $$\frac{{\left(\frac{\varepsilon^{2}}{\hbar^{2}}\Delta M+\Delta \Phi +\Sigma_{phe}\left(\varepsilon \right)+\Sigma_{phph}\left(\varepsilon \right)\right)}_{ni\alpha ,n^{\prime }i^{\prime }\alpha^{\prime }}}{\Phi_{ni\alpha ,n^{\prime }i^{\prime }\alpha^{\prime }}^{\left(0\right)}}\ll 1,$$

then the solution of the system of equations in Equation (45) becomes

(57) $$\begin{array}{ccc} G^{aa^{+}}\left(\varepsilon \right) & = & {\left[{\left[G_{0}^{aa^{+}}\left(\varepsilon \right)\right]}^{-1}-\left(w+\Sigma_{eph}\left(\varepsilon \right)+\Sigma_{ee}\left(\varepsilon \right)\right)\right]}^{-1}\\ G^{uu}\left(\varepsilon \right) & = & [{\left[G_{0}^{uu}\left(\varepsilon \right)\right]}^{-1}\\ & - & \left(\frac{\varepsilon^{2}}{\hbar^{2}}\Delta M+\Delta \Phi +\Sigma_{phe}\left(\varepsilon \right)+\Sigma_{phph}\left(\varepsilon \right)\right){]}^{-1}\\ G^{PP}\left(\varepsilon \right) & = & \frac{\varepsilon^{2}}{\hbar^{2}}{\left(M^{\left(0\right)}\right)}^{2}\;G^{uu}\left(\varepsilon \right) \end{array}$$

where

(58) $$\Delta M=∥\left(M_{i}-M_{ni}\right)\delta_{nn^{\prime }}\delta_{ii^{\prime }}\delta_{\alpha \alpha^{\prime }}∥.$$

These solutions only include the first-order corrections due to the small terms in Equation (56). Ref. [31] summarizes the explicit formulas for the corresponding self-energies, which we do not reproduce here.

**Figure 1:**
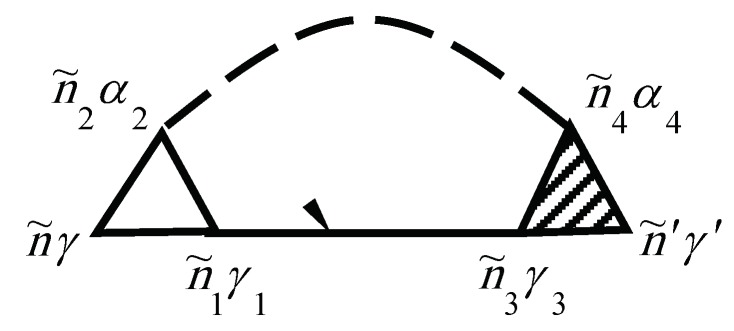
Diagram for the electron–phonon self-energy $\Sigma_{eph\;ni\gamma ,n^{\prime }i^{\prime }\gamma^{\prime }}\left(\tau ,\tau^{\prime }\right)=\Sigma_{eph\;\tilde{n}\gamma ,{\tilde{n}}^{\prime }\gamma^{\prime }}$. Here, $\tilde{n}=\left(ni\tau \right)$.

The electronic self-energy due to the electron–phonon interaction $\Sigma_{eph}\left(\tau ,\tau^{\prime }\right)$ is described by the diagram in Figure 1. The solid lines correspond to electronic propagators $G_{ni\gamma ,n^{\prime }i^{\prime }\gamma^{\prime }}^{aa^{+}}\left(\tau ,\tau^{\prime }\right)$ and the dashed lines correspond to phonon propagators $G_{ni\alpha ,n^{\prime }i^{\prime }\alpha^{\prime }}^{uu}\left(\tau ,\tau^{\prime }\right)$.

**Figure 2:**
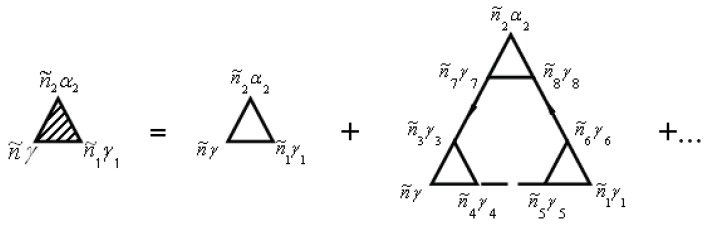
Diagrams for the vertex corrections $\Gamma_{ni\gamma ,n_{1}i_{1}\gamma_{1}}^{n_{2}i_{2}\alpha_{2}}\left(\tau_{2},\tau ,\tau_{1}\right)=\Gamma_{\tilde{n}\gamma ,{\tilde{n}}_{1}\gamma_{1}}^{{\tilde{n}}_{2}\alpha_{2}}$. Here, $\tilde{n}=\left(ni\tau \right)$.

The vertex correction $\Gamma_{ni\gamma ,n_{1}i_{1}\gamma_{1}}^{n_{2}i_{2}\alpha_{2}}\left(\tau_{2},\tau ,\tau_{1}\right)$ is given by the diagrams in Figure 2. Note that the unshaded triangle corresponds to the equation

(59) $$\Gamma_{0\;ni\gamma ,n{}^{\prime }i{}^{\prime }\gamma {}^{\prime }}^{n{}^{\prime \prime }i{}^{\prime \prime }\alpha {}^{\prime \prime }}\left(\tau {}^{\prime \prime },\tau ,\tau {}^{\prime }\right)={v{}^{\prime }}_{ni\gamma ,n{}^{\prime }i{}^{\prime }\gamma {}^{\prime }}^{n{}^{\prime \prime }i{}^{\prime \prime }\alpha {}^{\prime \prime }}\delta \left(\tau -\tau {}^{\prime \prime }\right)\;\delta \left(\tau -\tau {}^{\prime }\right).$$

In Figure 1 and Figure 2, the internal summations for $\tilde{n}\gamma$ imply both a summation over niγ and an integration over the internal time τ. Each diagram has an overall sign determined by ${\left(-1\right)}^{n+F}$, where *N* is the order of the diagram (number of vertices $\Gamma_{0})$, and *F* is the number of electronic Green’s function lines $G^{aa^{+}}$.

Explicitly, the electron–phonon self-energy becomes

(60) $$\begin{array}{c} \Sigma_{eph\;ni\gamma ,n^{\prime }i^{\prime }\gamma^{\prime }}\left(\varepsilon \right)\\ =-\frac{1}{4\pi i}\int_{-\infty }^{\infty }d\varepsilon^{\prime }\coth\left(\frac{\varepsilon^{\prime }}{2\Theta }\right){v^{\prime }}_{ni\gamma ,n_{3}i_{3}\gamma_{3}}^{n_{1}i_{1}\alpha_{1}}\\ \times \left[G_{n_{1}i_{1}\alpha_{1},n_{2}i_{2}\alpha_{2}}^{uu}\left(\varepsilon^{\prime }\right)-G_{n_{1}i_{1}\alpha_{1},n_{2}i_{2}\alpha_{2}}^{uu\;*}\left(\varepsilon^{\prime }\right)\right]\\ \times G_{n_{3}i_{3}\gamma_{3},n_{4}i_{4}\gamma_{4}}^{aa^{+}}\left(\varepsilon -\varepsilon^{\prime }\right)\\ \times \Gamma_{n_{4}i_{4}\gamma_{4},n^{\prime }i^{\prime }\gamma^{\prime }}^{n_{2}i_{2}\alpha_{2}}\left(\varepsilon -\varepsilon^{\prime };\varepsilon ;\varepsilon^{\prime }\right) \end{array}$$

where repeated indices are summed over.

**Figure 3:**
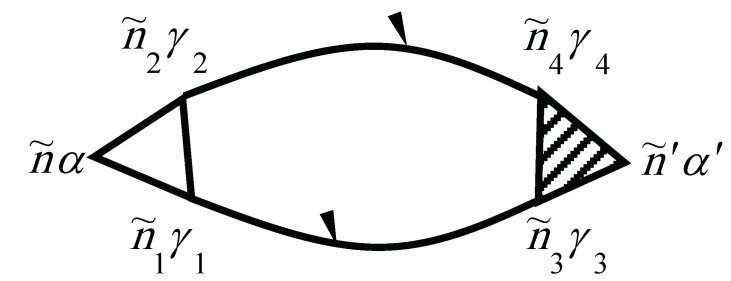
Diagram for $\Sigma_{phe\;ni\alpha ,n^{\prime }i^{\prime }\alpha^{\prime }}\left(\tau ,\tau^{\prime }\right)=\Sigma_{phe\;\tilde{n}\alpha ,{\tilde{n}}^{\prime }\alpha^{\prime }}$. Here, $\tilde{n}=\left(ni\tau \right)$.

**Figure 4:**
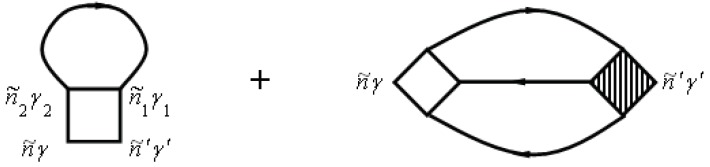
Diagrams for $\Sigma_{ee\;ni\gamma ,n^{\prime }i^{\prime }\gamma^{\prime }}\left(\tau ,\tau^{\prime }\right)=\Sigma_{ee\;\tilde{n}\gamma ,{\tilde{n}}^{\prime }\gamma^{\prime }}$. Here, $\tilde{n}=\left(ni\tau \right)$.

**Figure 5:**
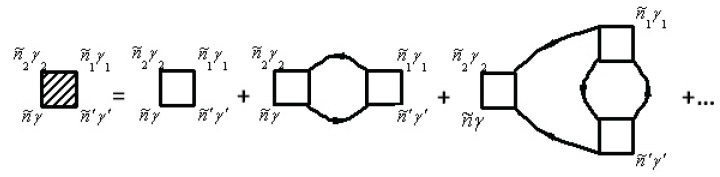
Diagrams for vertex part $\Gamma_{ni\gamma ,n^{\prime }i^{\prime }\gamma^{\prime }}^{n_{2}i_{2}\gamma_{2},n_{1}i_{1}\gamma_{1}}\left(\tau_{2},\tau_{1}\tau ,\tau^{\prime }\right)=\Gamma_{\tilde{n}\gamma ,{\tilde{n}}^{\prime }\gamma^{\prime }}^{{\tilde{n}}_{2}\gamma_{2},{\tilde{n}}_{1}\gamma_{1}}$. Here, $\tilde{n}=\left(ni\tau \right)$.

The self-energy of the phonon due to the phonon–electron interaction is shown in Figure 3. Evaluating the diagram using the above rules yields

(61) $$\begin{array}{c} \Sigma_{pheni\alpha ,n^{\prime }i^{\prime }\alpha^{\prime }}\left(\varepsilon \right)=\frac{1}{2\pi i}\int_{-\infty }^{\infty }d\varepsilon^{\prime }f\left(\varepsilon^{\prime }\right){v^{\prime }}_{n_{2}i_{2}\gamma_{2},n_{1}i_{1}\gamma_{1}}^{ni\alpha }\\ \times \left\{\left[G_{n_{1}i_{1}\gamma_{1},n_{3}i_{3}\gamma_{3}}^{aa^{+}}\left(\varepsilon +\varepsilon^{\prime }\right)-G_{n_{1}i_{1}\gamma_{1},n_{3}i_{3}\gamma_{3}}^{aa^{+}*}\left(\varepsilon +\varepsilon^{\prime }\right)\right]\right.\\ \times \;G_{n_{4}i_{4}\gamma_{4},n_{2}i_{2}\gamma_{2}}^{aa^{+}*}\left(\varepsilon^{\prime }\right)+G_{n_{1}i_{1}\gamma_{1},n_{3}i_{3}\gamma_{3}}^{aa^{+}}\left(\varepsilon +\varepsilon^{\prime }\right)\\ \times \left.\left[G_{n_{4}i_{4}\gamma_{4},n_{2}i_{2}\gamma_{2}}^{aa^{+}}\left(\varepsilon^{\prime }\right)-G_{n_{4}i_{4}\gamma_{4},n_{2}i_{2}\gamma_{2}}^{aa^{+}*}\left(\varepsilon^{\prime }\right)\right]\right\}\\ \times \;\Gamma_{n_{3}i_{3}\gamma_{3},n_{4}i_{4}\gamma_{4}}^{n^{\prime }i^{\prime }\alpha^{\prime }}\left(\varepsilon +\varepsilon^{\prime };\varepsilon^{\prime }\right). \end{array}$$

f(ε) is the so-called Fermi–Dirac distribution function. The electron–electron scattering contribution to the electronic self-energy $\Sigma_{ee}\left(\tau ,\tau^{\prime }\right)$ is shown in Figure 4, while the vertex part $\Gamma_{ni\gamma ,n^{\prime }i^{\prime }\gamma^{\prime }}^{n_{2}i_{2}\gamma_{2},n_{1}i_{1}\gamma_{1}}\left(\tau_{2},\tau_{1}\tau ,\tau^{\prime }\right)$ is given in Figure 5.

Note that the unshaded square in Figure 5 corresponds to the equation

(62) $$\begin{array}{c} \Gamma_{0\;ni\gamma ,n{}^{\prime }i{}^{\prime }\gamma {}^{\prime }}^{n{}^{\prime \prime \prime }i{}^{\prime \prime \prime }\gamma {}^{\prime \prime \prime },n{}^{\prime \prime }i{}^{\prime \prime }\gamma {}^{\prime \prime }}\left(\tau {}^{\prime \prime \prime },\tau {}^{\prime \prime }\tau ,\tau {}^{\prime }\right)={\tilde{v}}_{n{}^{\prime \prime }i{}^{\prime \prime }\gamma {}^{\prime \prime },n{}^{\prime }i{}^{\prime }\gamma {}^{\prime }}^{\left(2\right)ni\gamma ,n{}^{\prime \prime \prime }i{}^{\prime \prime \prime }\gamma {}^{\prime \prime \prime }}\delta \;\\ \times \;\left(\tau -\tau {}^{\prime \prime \prime }\right)\delta \left(\tau -\tau {}^{\prime \prime }\right)\delta \left(\tau -\tau {}^{\prime }\right)\\ {\tilde{v}}_{n{}^{\prime \prime }i{}^{\prime \prime }\gamma {}^{\prime \prime },n{}^{\prime }i{}^{\prime }\gamma {}^{\prime }}^{\left(2\right)ni\gamma ,n{}^{\prime \prime \prime }i{}^{\prime \prime \prime }\gamma {}^{\prime \prime \prime }}=v_{n{}^{\prime \prime }i{}^{\prime \prime }\gamma {}^{\prime \prime },n{}^{\prime }i{}^{\prime }\gamma {}^{\prime }}^{\left(2\right)ni\gamma ,n{}^{\prime \prime \prime }i{}^{\prime \prime \prime }\gamma {}^{\prime \prime \prime }}\;-v_{n{}^{\prime }i{}^{\prime }\gamma {}^{\prime },n{}^{\prime \prime }i{}^{\prime \prime }\gamma {}^{\prime \prime }}^{\left(2\right)ni\gamma ,n{}^{\prime \prime \prime }i{}^{\prime \prime \prime }\gamma {}^{\prime \prime \prime }}. \end{array}$$

Using this vertex function, then yields the contribution to the electron self-energy from the electron–electron interaction:

(63) $$\begin{array}{c} \Sigma_{ee\;ni\gamma ,n^{\prime }i^{\prime }\gamma^{\prime }}\left(\varepsilon \right)=\Sigma_{ee\;ni\gamma ,n^{\prime }i^{\prime }\gamma^{\prime }}^{\left(1\right)}+\Sigma_{ee\;ni\gamma ,n^{\prime }i^{\prime }\gamma^{\prime }}^{\left(2\right)}\left(\varepsilon \right)\\ \Sigma_{ee\;n,n^{\prime }}^{\left(1\right)}=-\frac{1}{4\pi i}\int_{-\infty }^{\infty }d\varepsilon^{\prime }\;f\left(\varepsilon^{\prime }\right)\;{\tilde{v}}_{\;n_{1},\;n^{\prime }}^{\left(2\right)n,n_{2}}\\ \times \left[G_{n_{1},n_{2}}^{aa^{+}}\left(\varepsilon^{\prime }\right)-G_{n_{1},n_{2}}^{aa^{+}\;*}\left(\varepsilon^{\prime }\right)\right]\\ \Sigma_{ee\;n,n^{\prime }}^{\left(2\right)}\left(\varepsilon \right)=-{\left(\frac{1}{2\pi i}\right)}^{2}\int_{-\infty }^{\infty }d\varepsilon_{1}\\ \times \int_{-\infty }^{\infty }d\varepsilon_{2}\;{\tilde{v}}_{n_{2},n_{1}}^{\left(2\right)n,n_{3}}\{f\left(\varepsilon_{1}\right)f\left(\varepsilon_{2}\right)\\ \times [G_{n_{2},n_{5}}^{aa^{+}\;*}\left(\varepsilon -\varepsilon_{1}-\varepsilon_{2}\right)G_{n_{1},n_{4}}^{aa^{+}}\left(\varepsilon_{1}\right)\\ -G_{n_{2},n_{5}}^{aa^{+}}\left(\varepsilon -\varepsilon_{1}-\varepsilon_{2}\right)G_{n_{1},n_{4}}^{aa^{+}\;*}\left(\varepsilon_{1}\right)]\\ \times \left[G_{n_{6},n_{3}}^{aa^{+}}\left(\varepsilon_{2}\right)-G_{n_{6},n_{3}}^{aa^{+}\;*}\left(\varepsilon_{2}\right)\right]\\ +\left[G_{n_{2},n_{5}}^{aa^{+}}\left(\varepsilon -\varepsilon_{1}-\varepsilon_{2}\right)-G_{n_{2},n_{5}}^{aa^{+}\;*}\left(\varepsilon -\varepsilon_{1}-\varepsilon_{2}\right)\right]\\ \times \left[G_{n_{1},n_{4}}^{aa^{+}}\left(\varepsilon_{1}\right)G_{n_{6},n_{3}}^{aa^{+}}\left(\varepsilon_{2}\right)-G_{n_{1},n_{4}}^{aa^{+}\;*}\left(\varepsilon_{1}\right)G_{n_{6},n_{3}}^{aa^{+}\;*}\left(\varepsilon_{2}\right)\right]\} \end{array}$$

(64) $$\begin{array}{c} \times \;\Gamma_{\;n_{4},\;n^{\prime }}^{\;n_{5},n_{6}}\left(\varepsilon -\varepsilon_{1}-\varepsilon_{2};\varepsilon_{2};\varepsilon_{1}\right) \end{array}$$

(65) $$\begin{array}{c} {\tilde{v}}_{n_{1},n^{\prime }}^{\left(2\right)n,n_{2}}=v_{n_{1},n^{\prime }}^{\left(2\right)n,n_{2}}-v_{n^{\prime },n_{1}}^{\left(2\right)n,n_{2}},\;\left(n\equiv ni\gamma \right). \end{array}$$

A similar result for the contribution to the phonon self-energy $\Sigma_{phph}\left(\varepsilon \right)$ from phonon–phonon coupling is given in [31]. In deriving the expressions in Equations (60), (61), and (64), we employed the standard resummation techniques for any function φ(z) that is analytic in the region covered by the contour *C*, which encloses all of the Matsubara frequencies. Namely, we have

(66) $$\begin{array}{c} \Theta \sum_{\omega_{n}}\varphi (i\omega_{n})=\frac{1}{4\pi i}\oint_{C}dz\;\coth\left(\frac{z}{2\Theta }\right)\varphi \left(z\right)\\ \left(\omega_{n}=2n\pi \Theta \right) \end{array}$$

for the Bosonic case, and

(67) $$\begin{array}{c} \Theta \sum_{\omega_{n}}\varphi (i\omega_{n})=-\frac{1}{2\pi i}\oint_{C}dz\;\tilde{f}\left(\frac{z}{\Theta }\right)\varphi \left(z\right)\\ \left(\omega_{n}=\left(2n+1\right)\pi \Theta \right) \end{array}$$

for the Fermionic case, with

(68) $$\tilde{f}\left(\frac{z}{\Theta }\right)=\frac{1}{\exp\left(\frac{z}{\Theta }\right)+1}.$$

We comment that, for the many-body Green’s functions described here, it is customary to have the chemical potential situated at zero frequency, as we do here.

In general, the renormalization of the vertex of the functions in Expressions (60), (61), and (64) for mass operators can be performed using Figure 2 and Figure 5. The first diagram in Figure 2 and Figure 5 corresponds to the equation

(69) $$\Gamma_{n_{4}i_{4}\gamma_{4},n^{\prime }i^{\prime }\gamma^{\prime }}^{\lambda_{2}n_{2}i_{2}\alpha_{2}}\left(\varepsilon -\varepsilon^{\prime };\varepsilon \right)={v^{\prime }}_{n_{4}i_{4}\gamma_{4},n^{\prime }i^{\prime }\gamma^{\prime }}^{\lambda_{2}n_{2}i_{2}\alpha_{2}}$$

and

(70) $$\Gamma_{n_{4},n^{\prime }}^{n_{5},n_{6}}\left(\varepsilon -\varepsilon_{1}-\varepsilon_{2};\varepsilon_{2};\varepsilon_{1}\right)={\tilde{v}}_{n_{6},n^{\prime }}^{\left(2\right)n_{4},n_{5}}.$$

The Fermi level $\varepsilon_{F}\equiv \mu_{e}$ of the system is determined by the equation

(71) $$\langle Z\rangle =\int_{-\infty }^{\infty }f\left(\varepsilon \right)\;g_{e}\left(\varepsilon \right)\;d\varepsilon ,\;f\left(\varepsilon \right)=\frac{1}{\exp(\frac{\varepsilon -\varepsilon_{F}}{\Theta })+1}$$

where 〈Z〉 is the average number of electrons per atom, and $g_{e}\left(\varepsilon \right)$ is the many-body electronic density of states, which satisfies

(72) ge(ε)=−1πνNImTrGaa+(ε)c.

Here, ${\langle \ldots \rangle }_{c}$ denotes configurational averaging over the disorder, *N* is the number of primitive lattice cells, and ν is the number of atoms per primitive cell. We drop the letter *c* on the configurational averaging for simplicity. In Equation (71), $\langle Z\rangle$ is the average number of electrons per atom.

It should be noted that the first term in the electron self-energy due to electron–electron interactions, $\Sigma_{ee\;ni\gamma ,n^{\prime }i^{\prime }\gamma^{\prime }}^{\left(1\right)}$ in Equation (59), describes the Coulomb and exchange electron–electron interactions in the Hartree–Fock approximation. The second term, $\Sigma_{ee\;ni\gamma ,n^{\prime }i^{\prime }\gamma^{\prime }}^{\left(2\right)}\left(\varepsilon \right)$, which is caused by corrections beyond Hartree–Fock, describes the effects of electron correlations. As opposed to the procedures used in [13,14,15,16,17,18,19], the long-range Coulomb interaction of electrons located at different lattices sites of the crystal is described by taking into account an arbitrary number of energy bands.

The expression for the Green’s function in Equation (54) differs from the corresponding expressions for the Green’s function of a single-particle Hamiltonian of a disordered system only from the different self-energy contributions. Hence, we solve for the Green’s function using the well-known methods of disordered systems theory [28].

## 4. Localized Magnetic Moments

Since we will be working with magnetic moments for the remainder of the paper, we now slightly modify our notation so that the symbol γ now refers to all other quantum numbers except for spin, and we will introduce the spin quantum number σ explicitly in all of the following equations. We will be employing the approximate results for the electron–electron self-energy in a self-consistent fashion to allow correlation effects to modify the electronic bands. This requires a heterogeneous distribution of the electron density. We assume that this electron density distribution corresponds to the minimum of the free energy. The electron–electron self-energy in Equation (64) requires the occupation number $Z_{ni\gamma \sigma }^{\lambda m_{\lambda i}}$ of the different electronic states (niγσ), where we are explicitly including the dependence on σ. The explicit values for $Z_{ni\gamma \sigma }^{\lambda m_{\lambda i}}$ are calculated from Equation (66), where the total electronic density of states $g_{e}\left(\varepsilon \right)$ is replaced by the partial density of states $g_{ni\gamma \sigma }^{\lambda m_{\lambda i}}\left(\varepsilon \right)$ for energy band γ and spin projection σ to allow for magnetic solutions. The occupation numbers $Z_{ni\gamma \sigma }^{\lambda m_{\lambda i}}$ and the partial density of states $g_{ni\gamma \sigma }^{\lambda m_{\lambda i}}\left(\varepsilon \right)$ then satisfy

(73) $$Z_{ni\gamma \sigma }^{\lambda m_{\lambda i}}=\int_{-\infty }^{\infty }f\left(\varepsilon \right)\;g_{ni\gamma \sigma }^{\lambda m_{\lambda i}}\left(\varepsilon \right)\;d\varepsilon$$

(74) gniγσλmλi(ε)=−1πImGniγσ,niγσaa+(ε)(ni)∈λmλi.

Note that the disorder averaging is done under the assumption that an atom of type λ is located at the site (ni), and its projection of the localized magnetic moment onto the *z*-axis is equal to $m_{\lambda i}$. The probability of this configuration is $P_{ni}^{\lambda m_{\lambda i}}$, and we have the obvious constraint that

(75) $$\sum_{\lambda ,m_{\lambda i}}P_{ni}^{\lambda m_{\lambda i}}=1.$$

In this fashion, we allow for localized magnetic moments which are inhomogeneously distributed through the crystal lattice and correspond to static magnetization fluctuations.

The total charge and magnetization for each orbital on a site are given by

(76) $$Z_{ni\gamma }^{\lambda m_{\lambda i}}=Z_{ni\gamma \sigma }^{\lambda m_{\lambda i}}+Z_{ni\gamma ,-\sigma }^{\lambda m_{\lambda i}},\;m_{\lambda i\gamma }=Z_{ni\gamma \sigma }^{\lambda m_{\lambda i}}-Z_{ni\gamma ,-\sigma }^{\lambda m_{\lambda i}}$$

and by

(77) $$Z_{ni\gamma \sigma }^{\lambda m_{\lambda i}}=\frac{Z_{ni\gamma }^{\lambda m_{\lambda i}}+m_{\lambda i\gamma }}{2},\;Z_{ni\gamma ,-\sigma }^{\lambda m_{\lambda i}}=\frac{Z_{ni\gamma }^{\lambda m_{\lambda i}}-m_{\lambda i\gamma }}{2},$$

respectively. We need to sum over all other quantum numbers to get the totals:

(78) $$Z_{ni}^{\lambda m_{\lambda i}}=\sum_{\gamma }Z_{ni\gamma }^{\lambda m_{\lambda i}},\;m_{\lambda i}=\sum_{\gamma }m_{\lambda i\gamma }.$$

Next, we calculate the phonon Green’s function in the coordinate basis $G^{uu}\left(\varepsilon \right)$ by solving Equation (57). Here, we employ the following procedure to take into account the heterogeneity: First, we work with the homogeneous system, which is pure and has no disorder. Then we introduce the disorder and compute its effects via a cluster expansion related to cluster expansions from the theory of alloys. Therefore, the zeroth approximation for the Green’s function is the Green’s function of the pure system given by $G^{aa^{+}}\left(\varepsilon \right)$, which we call the effective medium Green’s function. Since the system is pure for the effective medium, we compute the effective medium Green’s function via a Fourier transformation

(79) $$\begin{array}{ccc} {\tilde{G}}_{ni\gamma \sigma ,n^{\prime }i^{\prime }\gamma^{\prime }\sigma^{\prime }}^{aa^{+}}\left(\varepsilon \right) & = & \frac{1}{N}\sum_{k}{\left[\varepsilon -H^{\left(1\right)}\left(k\right)\right]}_{i\gamma \sigma ,i^{\prime }\gamma^{\prime }\sigma^{\prime }}^{-1}\\ & \times & e^{ik(r_{n}+\rho_{i}-r_{n^{\prime }}-\rho_{i^{\prime }})} \end{array}$$

where

(80) $$H^{\left(1\right)}\left(k\right)=H_{0}^{\left(1\right)}\left(k\right)+{\tilde{\Sigma }}_{eph}\left(k,\varepsilon \right)+{\tilde{\Sigma }}_{ee}\left(k,\varepsilon \right)+\sigma_{e}\left(k,\varepsilon \right).$$

*N* is the number of primitive unit cells, and $\sigma_{e}\left(k,\varepsilon \right)$ is the self-energy of the effective medium, also called the coherent potential. The coherent potentials are determined via the coherent potential approximation, which is described in detail below, with the coherent potential given in Equation (90).

We do a similar procedure for the effective medium phonon Green’s function, which satisfies

(81) $$\begin{array}{ccc} {\tilde{G}}_{ni\alpha ,n^{\prime }i^{\prime }\alpha^{\prime }}^{uu}\left(\varepsilon \right) & = & \frac{1}{N}\sum_{k}{\left[\omega^{2}M^{\left(0\right)}-\Phi \left(k\right)\right]}_{i\alpha ,i^{\prime }\alpha^{\prime }}^{-1}\\ & \times & e^{ik(r_{n}+\rho_{i}-r_{n^{\prime }}-\rho_{i^{\prime }})} \end{array}$$

where we have

(82) $$\Phi \left(k\right)=\Phi^{\left(0\right)}\left(k\right)+{\tilde{\Sigma }}_{phe}\left(k,\varepsilon \right)+{\tilde{\Sigma }}_{phph}\left(k,\varepsilon \right)+\sigma_{ph}\left(k,\varepsilon \right)$$

and the coherent potential satisfies Equation (91).

Note that the wavevector **k** varies within the first Brillouin zone. Furthermore, ${\tilde{\Sigma }}_{eph}\left(k,\varepsilon \right)$ is the Fourier transformation of the matrix $\Sigma_{eph\;ni\gamma ,n^{\prime }i^{\prime }\gamma^{\prime }}\left(\varepsilon \right)$ given in Equation (60) for which the terms ${v^{\prime }}_{ni\gamma ,\;n_{3}i_{3}\gamma_{3}}^{n_{1}i_{1}\alpha_{1}}$ are replaced by the values for a pure crystal and the corresponding Green’s functions are those of the effective medium. The other self-energies given by ${\tilde{\Sigma }}_{ee}\left(k,\varepsilon \right)$, ${\tilde{\Sigma }}_{phe}\left(k,\varepsilon \right)$, and ${\tilde{\Sigma }}_{phph}\left(k,\varepsilon \right)$ are defined similarly. In Equation (82), $\Phi^{\left(0\right)}\left(k\right)$ is the Fourier transform of the matrix $\Phi_{ni\alpha ,n^{\prime }i^{\prime }\alpha^{\prime }}^{\left(0\right)}$, which describes the atomic nucleus repulsion. The self-energy ${\tilde{\Sigma }}_{phe}\left(k,\varepsilon \right)$ describes the attractive interaction between the atomic nuclei and the electrons.

The Green’s functions in Equation (57) satisfy a Dyson equation that can be expressed in terms of a T-matrix via

(83) $$G\left(\varepsilon \right)=\tilde{G}\left(\varepsilon \right)+\tilde{G}\left(\varepsilon \right)\;T\left(\varepsilon \right)\;\tilde{G}\left(\varepsilon \right),$$

where the T-matrix *T* is represented by a series, in which each term describes the scattering of clusters with different numbers of nodes expressed schematically by

(84) $$T=\sum_{\left(n_{1}i_{1}\right)}t^{n_{1}i_{1}}+\sum_{\left(n_{1}i_{1}\right)\neq \left(n_{2}i_{2}\right)}T^{\left(2\right)\;n_{1}i_{1},n_{2}i_{2}}+\ldots \;.$$

Here, we have

(85) $$\begin{array}{ccc} T^{\left(2\right)n_{1}i_{1},n_{2}i_{2}} & = & {\left[I-t^{n_{1}i_{1}}\tilde{G}t^{n_{2}i_{2}}\tilde{G}\right]}^{-1}t^{n_{1}i_{1}}\\ & \times & \tilde{G}t^{n_{2}i_{2}}\left[I+\tilde{G}t^{n_{1}i_{1}}\right], \end{array}$$

where $t^{n_{1}i_{1}}$ is the on-site scattering operator given by

(86) $$t^{n_{1}i_{1}}={\left[I-\left(\Sigma^{n_{1}i_{1}}-\sigma^{n_{1}i_{1}}\right)\tilde{G}\right]}^{-1}\left(\Sigma^{n_{1}i_{1}}-\sigma^{n_{1}i_{1}}\right).$$

The self-energy employed in Equation (86), $\Sigma_{e}^{n_{1}i_{1}}\left(\varepsilon \right)$, satisfies

(87) $$w+\Sigma_{eph}\left(\varepsilon \right)+\Sigma_{ee}\left(\varepsilon \right)-{\tilde{\Sigma }}_{eph}\left(\varepsilon \right)-{\tilde{\Sigma }}_{ee}\left(\varepsilon \right)=\sum_{\left(n_{1}i_{1}\right)}\Sigma_{e}^{n_{1}i_{1}}\left(\varepsilon \right)$$

for the electrons. For the phonons, we have

(88) $$\begin{array}{c} \frac{\varepsilon^{2}}{\hbar^{2}}\Delta M+\Delta \Phi +\Sigma_{phe}\left(\varepsilon \right)+\Sigma_{phph}\left(\varepsilon \right)\\ -{\tilde{\Sigma }}_{phe}\left(\varepsilon \right)-{\tilde{\Sigma }}_{phph}\left(\varepsilon \right)=\sum_{\left(n_{1}i_{1}\right)}\Sigma_{ph}^{n_{1}i_{1}}\left(\varepsilon \right). \end{array}$$

The coherent potential approximation requires that

(89) $$\langle t^{0i_{1}}\rangle =0,$$

which yields a system of coupled equations [31] for the electronic

(90) $$\begin{array}{c} \sigma_{e}^{0i_{1}}\left(\varepsilon \right)={\langle {\left[1-\left(\Sigma_{e}^{0i_{1}}\left(\varepsilon \right)-\sigma_{e}^{0i_{1}}\left(\varepsilon \right)\right){\tilde{G}}^{aa^{+}}\left(\varepsilon \right)\right]}^{-1}\rangle }^{-1}\\ \times \langle {\left[1-\left(\Sigma_{e}^{0i_{1}}\left(\varepsilon \right)-\sigma_{e}^{0i_{1}}\left(\varepsilon \right)\right){\tilde{G}}^{aa^{+}}\left(\varepsilon \right)\right]}^{-1}\Sigma_{e}^{0i_{1}}\left(\varepsilon \right)\rangle \end{array}$$

and phononic

(91) $$\begin{array}{c} \sigma_{ph}^{0i_{1}}\left(\varepsilon \right)={\langle {\left[1-\left(\Sigma_{ph}^{0i_{1}}\left(\varepsilon \right)-\sigma_{ph}^{0i_{1}}\left(\varepsilon \right)\right){\tilde{G}}^{uu}\left(\varepsilon \right)\right]}^{-1}\rangle }^{-1}\\ \times \langle {\left[1-\left(\Sigma_{ph}^{0i_{1}}\left(\varepsilon \right)-\sigma_{ph}^{0i_{1}}\left(\varepsilon \right)\right){\tilde{G}}^{uu}\left(\varepsilon \right)\right]}^{-1}\Sigma_{ph}^{0i_{1}}\left(\varepsilon \right)\rangle \end{array}$$

coherent potentials.

Now we are ready to tackle the effects of disorder. Starting from Equation (83), we can obtain a cluster decomposition for the Green’s function of electrons and phonons. The electronic and phononic density of states, the free energy, and the electrical conductivity can all be expanded in an infinite series. Each term in the series describes the scattering on clusters with different numbers of atoms. It turns out that the strength of the scattering within a cluster decreases with an increasing number of atoms in the cluster and can be represented by the following small parameter:

(92) $$\begin{array}{c} p\left(\varepsilon \right)=\frac{1}{r\nu }\\ \times \left|\sum_{\left(n_{2}i_{2}\right)\neq \left(n_{1}i_{1}\right),i,\gamma }{\langle t^{n_{1}i_{1}}\left(\varepsilon \right)\tilde{G}\left(\varepsilon \right)t^{n_{2}i_{2}}\left(\varepsilon \right)\tilde{G}\left(\varepsilon \right)\rangle }_{0i\gamma ,0i\gamma }\right| \end{array}$$

where *r* is the total number of energy bands included in the calculation. We have shown previously [36,37] that this parameter remains small when many parameters of the system are changed, except possibly for narrow energy intervals near the band edges.

Next, we employ Equations (72), (74), and (83) to perform an average over the distribution of different types of atoms and different projections of the localized magnetic moments on the sites of the crystal lattice. During the averaging process, we neglect the contribution of electron scattering in clusters consisting of three or more atoms, since they are guaranteed to be small due to the smallness of the parameter in Equation (92). After performing the averaging, the electronic density of states becomes

(93) geε=1v∑i,γ,σ,λ,mλiP0iλmλig0iγσλmλi(ε),g0iγσλmλi(ε)=−1πIm{G˜+G˜t0iλmλiG˜+∑(lj)≠(0i)λ′,mλ′jPlj0iλ′mλ′j/λmλi×G˜tljλ′mλ′j+T(2)λmλi0i,λ′mλ′jljG˜}0iγσ,0iγσ,T(2)λmλi0i,λ′mλ′jlj=I−tλmλi0iG˜tλ′mλ′jljG˜−1×tλmλi0iG˜tλ′mλ′jljI+G˜tλmλi0i,

where $\tilde{G}={\tilde{G}}^{aa^{+}}\left(\varepsilon \right)$.

Similarly, averaging of the phonon Green’s function $G^{uu}\left(\varepsilon \right)$ yields the phononic density of states:

(94) gphε=1ν∑i,α,λP0iλg0iαλε,g0iαλ(ε)=−1π2εℏ2Mi×Im{G˜+G˜tλ0iG˜+∑(lj)≠(0i)λ′Plj0iλ′/λG˜[tλ′lj+T(2)λ0i,λ′lj]G˜}0iα,0iα,

where $\tilde{G}={\tilde{G}}^{uu}\left(\varepsilon \right)$.

In these formulas, the single-center scattering operator $t^{\lambda n_{1}i_{1}}$ is given by Equation (80). According to Equations (6), (60), (64), and (87), the self-energy denoted by $\Sigma_{e}^{\lambda n_{1}i_{1}}\left(\varepsilon \right)$ describes the electron scattering:

(95) $$\begin{array}{c} \Sigma_{eni\gamma \sigma ,n{}^{\prime }i{}^{\prime }\gamma {}^{\prime }\sigma {}^{\prime }}^{\lambda n{}^{\prime \prime }i{}^{\prime \prime }}\left(\varepsilon \right)=w_{ni\gamma \sigma ,n{}^{\prime }i{}^{\prime }\gamma {}^{\prime }\sigma {}^{\prime }}^{\lambda n{}^{\prime \prime }i{}^{\prime \prime }}\\ +\;\frac{1}{2}\;\sum_{\begin{array}{c} n{}^{\prime \prime }i{}^{\prime \prime }\gamma {}^{\prime \prime }\sigma {}^{\prime \prime }\\ n{}^{\prime \prime \prime }i{}^{\prime \prime \prime }\gamma {}^{\prime \prime \prime } \end{array}}\;{\tilde{v}}_{n{}^{\prime \prime }i{}^{\prime \prime }\gamma {}^{\prime \prime }\sigma {}^{\prime \prime },n{}^{\prime }i{}^{\prime }\gamma {}^{\prime }\sigma {}^{\prime }}^{\left(2\right)ni\gamma \sigma ,n{}^{\prime \prime \prime }i{}^{\prime \prime \prime }\gamma {}^{\prime \prime \prime }\sigma {}^{\prime \prime }}(Z_{n{}^{\prime \prime }i{}^{\prime \prime }\gamma {}^{\prime \prime }\sigma {}^{\prime \prime },n{}^{\prime \prime \prime }i{}^{\prime \prime \prime }\gamma {}^{\prime \prime \prime }\sigma {}^{\prime \prime }}^{\lambda m_{\lambda }}\\ -\;{\tilde{Z}}_{n{}^{\prime \prime }i{}^{\prime \prime }\gamma {}^{\prime \prime }\sigma {}^{\prime \prime },n{}^{\prime \prime \prime }i{}^{\prime \prime \prime }\gamma {}^{\prime \prime \prime }\sigma {}^{\prime \prime }}) \end{array}$$

where

(96) Zniγσ,n′i′γ′σ′λmλi=−1π∫−∞∞f(ε,εF)×ImGniγσ,n′i′γ′σ′aa+(ε)|(ni)∈λmλidε.

The value of ${\tilde{Z}}_{n_{1}i_{1}\gamma_{1}\sigma_{1},n_{2}i_{2}\gamma_{2}\sigma_{2}}$ in Equation (95) is derived from Equation (96) by replacing the full Green’s function by the effective medium Green’s function. The diagonal elements of the matrix $Z_{ni\gamma \sigma ,n^{\prime }i^{\prime }\gamma^{\prime }\sigma^{\prime }}^{\lambda m_{\lambda i}}$ in Equation (96) are equal to the occupation numbers of the electron states $Z_{ni\gamma \sigma }^{\lambda m_{\lambda i}}$ in Equation (73).

Similarly, according to Equations (9), (57), (61), and (88), the self-energy $\Sigma_{ph}^{\lambda n_{1}i_{1}}\left(\varepsilon \right)$ in Equation (86) describes phonon scattering,

(97) $$\begin{array}{ccc} \Sigma_{phni\alpha ,n^{\prime }i^{\prime }\alpha^{\prime }}^{\lambda n_{1}i_{1}}\left(\varepsilon \right) & = & \left[\frac{\varepsilon^{2}}{\hbar^{2}}\left(M_{i_{1}}-M_{\lambda }\right)\delta_{nn^{\prime }}\delta_{ii^{\prime }}\delta_{\alpha \alpha^{\prime }}\right.\\ & + & \left.\Phi_{ni\alpha ,n^{\prime }i^{\prime }\alpha^{\prime }}^{\lambda }-\Phi_{ni\alpha ,n^{\prime }i^{\prime }\alpha^{\prime }}^{\left(0\right)}\right]\delta_{nn_{1}}\delta_{ii_{1}}. \end{array}$$

In Equation (93), $P_{lj\;0\;i}^{\lambda^{\prime }m_{\lambda^{\prime }j}/\lambda \;m_{\lambda i}}$ is the conditional probability of finding an atom of type $\lambda^{\prime }$ at site (*lj*) with magnetic moment $m_{\lambda^{\prime }j},$ provided that the sites in the unit cell at the origin (0i) have an atom of type λ with a magnetic moment $m_{\lambda i}$. Here, $t_{ni}^{\lambda \;m_{\lambda i}}$ is the value of the matrix element of a single-center operator for scattering in the case where an atom of type λ is located at site (ni) and has a magnetic moment $m_{\lambda i}$.

When the system is disordered, we need to consider a random arrangement of the disordered atomic sites. Hence, in Equation (94), the probability of an atom of type λ to be at site (0i) is given by

(98) $$P_{0i}^{\lambda }=\langle c_{0i}^{\lambda }\rangle$$

where $c_{ni}^{\lambda }$ is a discrete binary random number taking the values of 1 or 0, depending on whether an atom of type λ is at site (ni) or not, respectively. The joint probabilities in Equations (86) and (94) are defined by the following:

$$P_{lj\;\;0i}^{\lambda^{\prime }\lambda }=P_{0i}^{\lambda }P_{lj\;0i}^{\lambda^{\prime }/\lambda }=\langle c_{lj}^{\lambda^{\prime }}c_{0i}^{\lambda }\rangle$$

$$P_{0i}^{\lambda \;m_{\lambda i}}=P_{0i}^{\lambda }P_{0i}^{m_{\lambda i}},\;\;P_{lj\;0i}^{\lambda^{\prime }m_{\lambda^{\prime }j}/\lambda m_{\lambda i}}=P_{lj\;0i}^{\lambda^{\prime }/\lambda }P_{lj\;0i}^{m_{\lambda^{\prime }j}/m_{\lambda i}}$$

(99) $$P_{lj\;0i}^{m_{\lambda^{\prime }j}\;m_{\lambda i}}=P_{0i}^{m_{\lambda i}}P_{lj\;0i}^{m_{\lambda^{\prime }j}/m_{\lambda i}}=\langle c_{lj}^{m_{\lambda^{\prime }j}}c_{0i}^{m_{\lambda i}}\rangle .$$

The probabilities are determined by the interatomic pair correlations $\varepsilon_{lj\;0i}^{\;B\;B}$, $\varepsilon_{\;lj\;0i}^{\;\mu_{\lambda^{\prime }j}^{-}\;\mu_{\lambda i}^{-}}$ via [23,27]

(100) $$\begin{array}{c} P_{lj\;0i}^{\lambda^{\prime }/\lambda }=P_{lj}^{\lambda^{\prime }}+\frac{\varepsilon_{lj\;0i}^{B\;B}}{P_{0i}^{\lambda }}\left(\delta_{\lambda^{\prime }B}-\delta_{\lambda^{\prime }A}\right)\left(\delta_{\lambda B}-\delta_{\lambda A}\right)\\ P_{lj\;0i}^{m_{\lambda^{\prime }j}/m_{\lambda i}}=P_{lj}^{m_{\lambda^{\prime }j}}+\frac{\varepsilon_{lj\;0i}^{\mu_{\lambda^{\prime }j}^{-}\mu_{\lambda i}^{-}}}{P_{0i}^{m_{\lambda i}}}(\delta_{m_{\lambda^{\prime }j},\mu_{j}^{-}}\\ -\;\delta_{m_{\lambda^{\prime }j},\mu_{j}^{+}})\left(\delta_{m_{\lambda i},\mu_{i}^{-}}-\delta_{m_{\lambda i},\mu_{i}^{+}}\right) \end{array}$$

where δ is the Kronecker delta function. Note that the interatomic pair correlations also satisfy

(101) $$\begin{array}{ccc} \varepsilon_{lj\;\;0i}^{BB} & = & \langle \left(c_{lj}^{B}-c_{j}^{B}\right)\left(c_{0i}^{B}-c_{i}^{B}\right)\rangle ,\\ \varepsilon_{lj\;0i}^{\mu_{\lambda^{\prime }j}^{-}\mu_{\lambda i}^{-}} & = & \langle \left(c_{lj}^{\mu_{\lambda^{\prime }j}^{-}}-c_{j}^{\mu_{\lambda^{\prime }j}^{-}}\right)\left(c_{0i}^{\mu_{\lambda i}^{-}}-c_{i}^{\mu_{\lambda i}^{-}}\right)\rangle . \end{array}$$

The notations $P_{0i}^{m_{\lambda i}}$, $P_{lj\;0i}^{m_{\lambda^{\prime }j}/m_{\lambda i}}$ denote the probabilities of the static fluctuations of the magnetization.

As an example, when we have a binary alloy, consisting of two sublattices, and two types of atoms *A* and *B*, we obtain

(102) $$P_{0i}^{A}=x_{A}-\frac{\nu_{2}}{\nu }\eta_{a}$$

for the first sublattice and

(103) $$P_{0i}^{A}=x_{A}+\frac{\nu_{1}}{\nu }\eta_{a}$$

for the second sublattice, with

(104) $$P_{0i}^{B}=1-P_{0i}^{A}.$$

Here, we have $\nu =\nu_{1}+\nu_{2}$ is the total number of (given by more atoms of one type on Sublattice 1 and *vice versa* on Sublattice 2).

We assume that the projections of the localized magnetic moment onto the *z* axis are given by two values $m_{\lambda i}=\mu_{\lambda i}^{+}$, $\mu_{\lambda i}^{-}$. The probability $P_{0i}^{m_{\lambda i}}$ is connected with the long-range magnetic parameter $\eta_{m}$ via the expressions

(105) $$P_{0i}^{\mu_{\lambda i}^{+}}=x_{\mu_{\lambda }^{+}}-\frac{\nu_{2}}{\nu }\eta_{m}$$

for Sublattice 1 and

(106) $$P_{0i}^{\mu_{\lambda i}^{+}}=x_{\mu_{\lambda }^{+}}+\frac{\nu_{1}}{\nu }\eta_{m}$$

for Sublattice 2, with

(107) $$P_{0i}^{\mu_{\lambda i}^{-}}=1-P_{0i}^{\mu_{\lambda i}^{+}}.$$

Here, $x_{\mu_{\lambda }^{+}}$ and $x_{\mu_{\lambda }^{-}}=1-x_{\mu_{\lambda }^{+}}$ are equal to the relative number of lattice sites with localized magnetic moment projections $\mu_{\lambda i}^{+}$ and $\mu_{\lambda i}^{-}$, respectively. The value $x_{\mu_{\lambda }^{+}}=x_{\mu_{\lambda }^{-}}=0.5$ when the external magnetic field vanishes H=0, corresponding to a paramagnetic state.

So far, we have described how one performs one iteration of the self-consistent calculation. Once one iteration has been completed, the values of the occupation numbers of the electron states in Equation (16) are determined by Equation (73):

(108) $$\begin{array}{c} Z_{ni\gamma }^{\lambda }=\int_{-\infty }^{\infty }f\left(\varepsilon \right)\;g_{ni\delta \sigma }^{\lambda m_{\lambda i}}\left(\varepsilon \right)\;d\varepsilon \\ \delta =\left(\tilde{\varepsilon }lm\right) \end{array}.$$

The iterations continue until the densities of states have converged. Once convergence has been reached, we can then employ the Green’s functions to calculate observables, which is described in the next two sections.

## 5. Free Energy

We first focus on the Gibbs free energy (also called the thermodynamic potential) of the system which satisfies [32]:

(109) $$\Omega =-\Theta \;\ln\mathrm{Tr}(e^{-H/\Theta }).$$

The Hamiltonian *H* is defined in Equation (1). To perform the trace, we need to sum over all of the band states, but we also need to take into account the disorder averaging. The latter is commonly handled via a configurational average [31]. As for the correlations, we employ Equation (40) for the interaction picture, which allows us to re-express the Gibbs free energy as

(110) $$\Omega =\langle \delta \Phi \rangle -\Theta \;S_{c}+\Omega_{e}^{\left(0\right)}+\Omega_{ph}^{\left(0\right)}+\Omega^{\prime }$$

where $\Omega_{e}^{\left(0\right)}$ and $\Omega_{ph}^{\left(0\right)}$ are the thermodynamic potentials for the electrons and the phonons in the field of the ionic cores, respectively. As before, the equilibrium ion core positions are chosen to be the same as those of the crystal lattice for the pure ordered crystal, even when we introduce disorder and change the type of some of the atoms. The symbol $\Omega^{\prime }$ is the component of the thermodynamic potential that is caused by the mutual scattering of electrons and phonons; it is defined by

(111) $$\Omega^{\prime }=-\Theta \;\ln\langle {\langle \sigma \left(1/\Theta \right)\rangle }_{0}\rangle$$

with σ given in Equation (40) for the interaction picture.

In addition, $S_{c}=-\langle \ln P_{c}\rangle$ is the configurational entropy, where $P_{c}$ denotes the distribution function for atoms with a specific *z*-component of the magnetic moment on a given lattice site. The angular brackets $\langle \ldots \rangle$ denote the configurational averaging over different disorder configurations for a given density of disorder.

Next, we use the “integration over the coupling constant” method to simplify the results further. By replacing the interacting Hamiltonian $H_{\mathrm{int}}$ (defined in Equation (5)) by $H_{\mathrm{int}}\left(\lambda \right)=\lambda H_{\mathrm{int}}$, differentiating the expression for the piece of the thermodynamic potential $\Omega^{\prime }\left(\lambda \right)$ in Equation (111) with respect to λ, and then integrating them (with the boundary conditions $\Omega^{\prime }\left(0\right)=0$, $\Omega^{\prime }\left(1\right)=\Omega^{\prime })$, we obtain the following after a long derivation:

(112) Ω′=−1πνNIm∫01dλλ∫−∞∞dε[f(ε)×Trw(λ)+Σeph(ε,λ)+Σee(ε,λ)Gaa+(ε,λ)+12cothε2ΘTr〈ΔM−1(λ)GPP(ε,λ)+ΔΦ(λ)+Σphph(ε,λ)Guu(ε,λ)〉].

This expression can be immediately evaluated, because we know all the Green’s functions and self-energies.

The contribution to the thermodynamic potential from the electrons (in the field of the ionic cores) is also simple to find. It is given by

(113) $$\Omega_{e}^{\left(0\right)}=-\Theta \int_{-\infty }^{\infty }\ln\left(1+e^{(\mu_{e}-\varepsilon )/\Theta }\right)\;g_{e}^{\left(0\right)}\left(\varepsilon \right)\;d\varepsilon .$$

Similarly, the contribution to the thermodynamic potential from the phonons (in the field of the ionic cores) is given by

(114) $$\Omega_{ph}^{\left(0\right)}=\Theta \int_{-\infty }^{\infty }\ln\left(1-e^{-\varepsilon /\Theta }\right)\;g_{ph}^{\left(0\right)}\left(\varepsilon \right)\;\varepsilon .$$

The densities of states $g_{e}^{\left(0\right)}\left(\varepsilon \right)$, $g_{ph}^{\left(0\right)}\left(\varepsilon \right)$ in Equations (113) and (114) are given by the results in Equations (93) and (94); note that the Green’s functions $G^{aa^{+}}\left(\varepsilon \right)$ and $G^{uu}\left(\varepsilon \right)$ are replaced by Green’s functions $G_{0}^{aa^{+}}\left(\varepsilon \right)$ and $G_{0}^{uu}\left(\varepsilon \right)$ for the zeroth-order approximation.

Finally, the configurational entropy can be represented as [31]

(115) $$\begin{array}{c} S_{c}=-[\sum_{\lambda_{,}m_{\lambda i},ni}P_{ni}^{\lambda m_{\lambda i}}\ln P_{ni}^{\lambda m_{\lambda i}}\\ +\;\frac{1}{2}\;\sum_{\begin{array}{c} \lambda ,m_{\lambda i},ni,\\ \lambda^{\prime },m_{\lambda^{\prime }j},lj\\ \left(ni)\neq (lj\right) \end{array}}\;P_{nilj}^{\lambda m_{\lambda i}\lambda^{\prime }m_{\lambda^{\prime }j}}\ln\frac{P_{nilj}^{\lambda m_{\lambda i}\lambda^{\prime }m_{\lambda^{\prime }j}}}{P_{ni}^{\lambda m_{\lambda i}}P_{lj}^{\lambda^{\prime }m_{\lambda^{\prime }j}}}+\ldots ]. \end{array}$$

Ultimately, we are interested in determining the Helmholz free energy, *F*, as a function of the volume *V*, the temperature *T*, the number of electrons $N_{e}$, and the parameters of the interatomic correlations $\left(\varepsilon_{n_{1}i_{1},n_{2}i_{2}},\eta \right)$. The Helmholz free energy can be found directly from the thermodynamic potential. Namely, it satisfies $F=\Omega +\mu_{e}\langle N_{e}\rangle$. The free energy per atom can be approximated by [31]

(116) $$F=\langle \delta \Phi \rangle -\Theta \;S_{c}+\Omega_{e}+\Omega_{ph}+\mu_{e}\langle Z\rangle$$

where $\Omega_{e}$, $\Omega_{ph}$ are given by Equations (113) and (114), but with $g_{e}^{\left(0\right)}\left(\varepsilon \right)$ and $g_{ph}^{\left(0\right)}\left(\varepsilon \right)$ replaced by $g_{e}\left(\varepsilon \right)$ and $g_{ph}\left(\varepsilon \right)$ (see Equations (93) and (94)), in the situation where the electron scattering is weak.

## 6. Electrical Conductivity

In this section, we discuss how to calculate the electrical conductivity. We assume the system will not be driven too far from equilibrium, so we employ the linear response formalism of Kubo for the electrical conductivity tensor [38,39], which is given by

(117) $$\sigma_{\alpha \beta }\left(\omega \right)=\int_{0}^{1/\Theta }\int_{0}^{\infty }e^{i\omega t-\delta t}\langle {\tilde{J}}_{\beta }\left(0\right){\tilde{J}}_{\alpha }\left(t+i\hbar \tau \right)\rangle d\tau dt.$$

In this equation, $J_{\alpha }$ is the current operator along the α spatial direction. The real part of the conductivity, called the optical conductivity, can then be represented in terms of the imaginary part of the retarded response function, or equivalently as

(118) Reσαβω=i2ωGrJαJβω−GaJαJβω

in terms of the retarded and advanced response functions. The current operator is just the number operator for the electrons, multiplied by their velocity and the electric charge, and then summed over all states. It is compactly represented via

(119) $$J_{\alpha }\left(t\right)=e\int \Psi^{+}\left(\xi ,t\right)v_{\alpha }\Psi \left(\xi ,t\right)d\xi$$

where $\Psi^{+}\left(\xi ,t\right)$ and $\Psi \left(\xi ,t\right)$ are the field operators for the creation and annihilation of electrons, respectively, $\nu_{\alpha }$ is the operator of the α component of the band velocity, and *e* is the electron charge. The integration over ξ sums over all states.

To get the retarded response function on the real frequency axis, we must analytically continue the thermal response functions. The thermal current-current response function is defined to be

(120) $$\begin{array}{ccc} G^{J_{\alpha }J_{\beta }}\left(\tau ,\tau^{\prime }\right) & = & \frac{e^{2}}{NV_{1}}\sum_{n_{1}n_{2}n_{3}n_{4}}v_{\alpha n_{4}n_{2}}v_{\beta n_{3}n_{1}}\\ & \times & G{}^{\prime \prime }\left(n_{1}\tau^{\prime },n_{2}\tau ,n_{3}\tau^{\prime },n_{4}\tau \right) \end{array}$$

where $V_{1}$ is the volume of the primitive unit cell and the two-particle thermal Green’s function is given by the following time-ordered expectation value:

(121) $$\begin{array}{c} G{}^{\prime \prime }\left(n_{1}\tau^{\prime },n_{2}\tau ,n_{3}\tau^{\prime },n_{4}\tau \right)\\ =\langle T_{\tau }a_{n_{1}}\left(\tau^{\prime }\right)a_{n_{2}}\left(\tau \right)a_{n_{3}}^{+}\left(\tau^{\prime }\right)a_{n_{4}}^{+}\left(\tau \right)\\ \times \;\sigma \left(1/\theta \right)\rangle_{0}{\langle \sigma \left(1/\theta \right)\rangle }_{0}^{-1}\\ \left(n=ni\gamma \right). \end{array}$$

**Figure 6:**
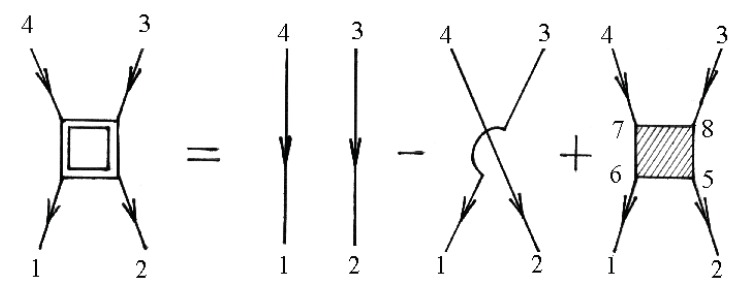
Diagrams for the two-particle Green’s function.

The two-particle Green’s function consists of a bare direct, a bare exchange, and a vertex-corrected piece, which are illustrated schematically in Figure 6.

The numbers at the vertices are a shortcut for all of the relevant quantum numbers and imaginary time, e.g., 1 corresponds to $\left(n_{1}i_{1}\gamma_{1}\tau_{1}\right)$.

Employing the diagram technique outlined above and in [31], and neglecting contributions to electron scattering on clusters of three or more sites, yields the following for the dc conductivity (ω→0):

(122) $$\begin{array}{c} \sigma_{\alpha \beta }=\frac{e^{2}\hbar }{4\pi V_{1}}\{\int_{-\infty }^{\infty }d\varepsilon_{1}\frac{\partial f}{\partial \varepsilon_{1}}\sum_{s,s^{\prime }=+,-}\left(2\delta_{ss^{\prime }}-1\right)\\ \times \sum_{\sigma \gamma ,i}\{\left[v_{\beta }\tilde{K}\left(\varepsilon_{1}^{s},v_{\alpha },\varepsilon_{1}^{s^{\prime }}\right)\right]\\ +\sum_{\lambda ,m_{\lambda i}}P_{0i}^{\lambda m_{\lambda i}}\tilde{K}\left(\varepsilon_{1}^{s^{\prime }},v_{\beta },\varepsilon_{1}^{s}\right)(t_{0i}^{\lambda m_{\lambda i}}\left(\varepsilon_{1}^{s}\right)\\ \times \;\tilde{K}\left(\varepsilon_{1}^{s},v_{\alpha },\varepsilon_{1}^{s^{\prime }}\right)t_{0i}^{\lambda m_{\lambda i}}\left(\varepsilon_{1}^{s^{\prime }}\right)\\ +\sum_{\lambda ,m_{\lambda i}}P_{0i}^{\lambda m_{\lambda i}}\sum_{\begin{array}{c} lj\neq 0i,\\ \lambda^{\prime },m_{\lambda^{\prime }j} \end{array}}P_{lj\;\;\;0i}^{\lambda^{\prime }m_{\lambda^{\prime }j}/\lambda m_{\lambda i}}\\ \times \;[[\tilde{K}\left(\varepsilon_{1}^{s^{\prime }},v_{\beta },\varepsilon_{1}^{s}\right)v_{\alpha }\tilde{G}\left(\varepsilon_{1}^{s^{\prime }}\right)]\\ \times \;T^{\left(2\right)\lambda m_{\lambda i}\;0i,\lambda^{\prime }m_{\lambda^{\prime }j}lj}\left(\varepsilon_{1}^{s^{\prime }}\right)\\ +\;\left[\tilde{K}\left(\varepsilon_{1}^{s^{\prime }},v_{\beta },\varepsilon_{1}^{s}\right)v_{\alpha }\tilde{G}\left(\varepsilon_{1}^{s^{\prime }}\right)\right]T^{\left(2\right)\lambda^{\prime }m_{\lambda^{\prime }j}lj,\lambda m_{\lambda i}\;0i}\left(\varepsilon_{1}^{s^{\prime }}\right)\\ +\;\left[\tilde{K}\left(\varepsilon_{1}^{s},v_{\alpha },\varepsilon_{1}^{s^{\prime }}\right)v_{\beta }\tilde{G}\left(\varepsilon_{1}^{s}\right)\right]T^{\left(2\right)\lambda \;m_{\lambda i}0i,\lambda^{\prime }m_{\lambda^{\prime }j}lj}\left(\varepsilon_{1}^{s}\right)\\ +\;\left[\tilde{K}\left(\varepsilon_{1}^{s},v_{\alpha },\varepsilon_{1}^{s^{\prime }}\right)v_{\beta }\tilde{G}\left(\varepsilon_{1}^{s}\right)\right]T^{\left(2\right)\lambda^{\prime }m_{\lambda^{\prime }j}lj,\lambda m_{\lambda i}\;0i}\left(\varepsilon_{1}^{s}\right)\\ +\;\tilde{K}\left(\varepsilon_{1}^{s^{\prime }},v_{\beta },\varepsilon_{1}^{s}\right)[t_{lj}^{\lambda^{\prime }m_{\lambda^{\prime }j}}\left(\varepsilon_{1}^{s}\right)\tilde{K}\left(\varepsilon_{1}^{s},v_{\alpha },\varepsilon_{1}^{s^{\prime }}\right)t_{0i}^{\lambda m_{\lambda i}}\left(\varepsilon_{1}^{s^{\prime }}\right)\\ +\;\left(t_{0i}^{\lambda m_{\lambda^{\prime }j}}\left(\varepsilon_{1}^{s}\right)+t_{lj}^{\lambda^{\prime }m_{\lambda^{\prime }j}}\left(\varepsilon_{1}^{s}\right)\right)\tilde{K}\left(\varepsilon_{1}^{s},v_{\alpha },\varepsilon_{1}^{s^{\prime }}\right)\\ \times \;T^{\left(2\right)\lambda \;m_{\lambda i}0i,\lambda^{\prime }m_{\lambda^{\prime }j}lj}\left(\varepsilon_{1}^{s^{\prime }}\right)\\ +\;T^{\left(2\right)\lambda^{\prime }m_{\lambda^{\prime }j}lj,\lambda m_{\lambda i}\;0i}\left(\varepsilon_{1}^{s}\right)\tilde{K}\left(\varepsilon_{1}^{s},v_{\alpha },\varepsilon_{1}^{s^{\prime }}\right)t_{0i}^{\lambda m_{\lambda i}}\left(\varepsilon_{1}^{s^{\prime }}\right)\\ +\;T^{\left(2\right)\lambda^{\prime }m_{\lambda^{\prime }j}lj,\lambda m_{\lambda i}\;0i}\left(\varepsilon_{1}^{s}\right)\tilde{K}\left(\varepsilon_{1}^{s},v_{\alpha },\varepsilon_{1}^{s^{\prime }}\right)\\ \times \;T^{\left(2\right)\lambda m_{\lambda i}\;0i,\lambda^{\prime }m_{\lambda^{\prime }j}lj}\left(\varepsilon_{1}^{s^{\prime }}\right)\\ +\;T^{\left(2\right)\lambda^{\prime }m_{\lambda^{\prime }j}lj,\lambda m_{\lambda i}\;0i}\left(\varepsilon_{1}^{s}\right)\tilde{K}\left(\varepsilon_{1}^{s},v_{\alpha },\varepsilon_{1}^{s^{\prime }}\right)\\ \times \;T^{\left(2\right)\lambda^{\prime }m_{\lambda^{\prime }j}lj,\lambda m_{\lambda i}\;0i}\left(\varepsilon_{1}^{s^{\prime }}\right)]]{\}}^{0i\gamma \sigma ,0i\gamma \sigma }\\ +\int_{-\infty }^{\infty }\int_{-\infty }^{\infty }d\varepsilon_{1}d\varepsilon_{2}f\left(\varepsilon_{1}\right)f\left(\varepsilon_{2}\right)\langle \Delta G_{\alpha \beta }^{II}\left(\varepsilon_{1},\varepsilon_{2}\right)\rangle \} \end{array}$$

where

(123) $$\begin{array}{c} \tilde{K}\left(\varepsilon_{1}^{s},v_{\alpha },\varepsilon_{1}^{s^{\prime }}\right)={\tilde{G}}^{aa^{+}}\left(\varepsilon_{1}^{s}\right)v_{\alpha }{\tilde{G}}^{aa^{+}}\left(\varepsilon_{1}^{s^{\prime }}\right)\\ {\tilde{G}}^{aa^{+}}\left(\varepsilon_{1}^{+}\right)={\tilde{G}}_{r}^{aa^{+}}\left(\varepsilon_{1}\right)\\ {\tilde{G}}^{aa^{+}}\left(\varepsilon_{1}^{-}\right)={\tilde{G}}_{a}^{aa^{+}}\left(\varepsilon_{1}\right)={\left({\tilde{G}}_{r}^{aa^{+}}\right)}^{*}\left(\varepsilon_{1}\right). \end{array}$$

The two-particle interaction term denoted by $\Delta G_{\alpha \beta }^{II}\left(\varepsilon_{1},\varepsilon_{2}\right)$ is given by

(124) $$\begin{array}{c} \Delta G_{\alpha \beta }^{II}\left(\varepsilon_{1},\varepsilon_{2}\right)=\frac{i}{2\pi }v_{\alpha n_{4}n_{2}}v_{\beta n_{3}n_{1}}\{[G_{rn_{1}n_{6}}^{aa^{+}}\left(\varepsilon_{1}\right)\\ -\;G_{an_{1}n_{6}}^{aa^{+}}\left(\varepsilon_{1}\right)]\left[G_{rn_{2}n_{5}}^{aa^{+}}\left(\varepsilon_{2}\right)-G_{an_{2}n_{5}}^{aa^{+}}\left(\varepsilon_{2}\right)\right]\\ \times \left[G_{an_{7}n_{4}}^{aa^{+}}\left(\varepsilon_{2}\right)G_{rn_{8}n_{3}}^{aa^{+}}\left(\varepsilon_{1}\right)-G_{rn_{7}n_{4}}^{aa^{+}}\left(\varepsilon_{2}\right)G_{an_{8}n_{3}}^{aa^{+}}\left(\varepsilon_{1}\right)\right]\\ +\;G_{an_{1}n_{6}}^{aa^{+}}\left(\varepsilon_{1}\right)\left[G_{rn_{2}n_{5}}^{aa^{+}}\left(\varepsilon_{2}\right)-G_{an_{2}n_{5}}^{aa^{+}}\left(\varepsilon_{2}\right)\right]\\ \times \;G_{an_{7}n_{4}}^{aa^{+}}\left(\varepsilon_{2}\right)\left[G_{rn_{8}n_{3}}^{aa^{+}}\left(\varepsilon_{1}\right)-G_{an_{8}n_{3}}^{aa^{+}}\left(\varepsilon_{1}\right)\right]\\ -\;G_{rn_{1}n_{6}}^{aa^{+}}\left(\varepsilon_{1}\right)\left[G_{rn_{2}n_{5}}^{aa^{+}}\left(\varepsilon_{2}\right)-G_{an_{2}n_{5}}^{aa^{+}}\left(\varepsilon_{2}\right)\right]\\ \times \;G_{rn_{7}n_{4}}^{aa^{+}}\left(\varepsilon_{2}\right)\left[G_{rn_{8}n_{3}}^{aa^{+}}\left(\varepsilon_{1}\right)-G_{an_{8}n_{3}}^{aa^{+}}\left(\varepsilon_{1}\right)\right]\\ +\;\left[G_{an_{1}n_{6}}^{aa^{+}}\left(\varepsilon_{1}\right)G_{rn_{2}n_{5}}^{aa^{+}}\left(\varepsilon_{2}\right)-G_{rn_{1}n_{6}}^{aa^{+}}\left(\varepsilon_{1}\right)G_{an_{2}n_{5}}^{aa^{+}}\left(\varepsilon_{2}\right)\right]\\ \times \;\left[G_{rn_{7}n_{4}}^{aa^{+}}\left(\varepsilon_{2}\right)-G_{an_{7}n_{4}}^{aa^{+}}\left(\varepsilon_{2}\right)\right]\\ \times \;\left[G_{rn_{8}n_{3}}^{aa^{+}}\left(\varepsilon_{1}\right)-G_{an_{8}n_{3}}^{aa^{+}}\left(\varepsilon_{1}\right)\right]\\ +\;\left[G_{rn_{1}n_{6}}^{aa^{+}}\left(\varepsilon_{1}\right)-G_{an_{1}n_{6}}^{aa^{+}}\left(\varepsilon_{1}\right)\right]\\ \times \;G_{rn_{2}n_{5}}^{aa^{+}}\left(\varepsilon_{2}\right)\left[G_{rn_{7}n_{4}}^{aa^{+}}\left(\varepsilon_{2}\right)-G_{an_{7}n_{4}}^{aa^{+}}\left(\varepsilon_{2}\right)\right]\\ \times \;G_{rn_{8}n_{3}}^{aa^{+}}\left(\varepsilon_{1}\right)-\left[G_{rn_{1}n_{6}}^{aa^{+}}\left(\varepsilon_{1}\right)-G_{an_{1}n_{6}}^{aa^{+}}\left(\varepsilon_{1}\right)\right]\\ \times \;G_{an_{2}n_{5}}^{aa^{+}}\left(\varepsilon_{2}\right)\left[G_{rn_{7}n_{4}}^{aa^{+}}\left(\varepsilon_{2}\right)-G_{an_{7}n_{4}}^{aa^{+}}\left(\varepsilon_{2}\right)\right]\\ \times \;G_{an_{8}n_{3}}^{aa^{+}}\left(\varepsilon_{1}\right)\}\Gamma_{n_{5}n_{8}}^{n_{6}n_{7}}\left(\varepsilon_{2};\varepsilon_{1};\varepsilon_{2}\right). \end{array}$$

n≡niγσ. The electron velocity satisfies the conventional definition

(125) $$v_{\alpha }\left(k\right)=\frac{1}{\hbar }\frac{\partial H_{0}^{\left(1\right)}\left(k\right)}{\partial k_{\alpha }}.$$

The interaction piece is evaluated approximately via $\langle \Delta G_{\alpha \beta }^{II}\left(\varepsilon_{1};\varepsilon_{2}\right)\rangle \approx \Delta {\tilde{G}}_{\alpha \beta }^{II}\left(\varepsilon_{1};\varepsilon_{2}\right)$, where $\Delta {\tilde{G}}_{\alpha \beta }^{II}\left(\varepsilon_{1};\varepsilon_{2}\right)$ is given in Equation (124) but replacing $G^{aa^{+}}\left(\varepsilon \right)$ with ${\tilde{G}}^{aa^{+}}\left(\varepsilon \right)$.

The above derivation yields the dc conductivity in the presence of a static electric field. In this work, we are also interested in the effects of a weak external magnetic field. Within a nonrelativistic approximation, an external magnetic field is introduced into the kinetic energy of an electron via the matrix element $h_{n_{1}i_{1}\gamma_{1},n_{2}i_{2}\gamma_{2}}^{\left(0\right)}$ (see Equation (3)). This is done using the minimal substitution ${\hat{p}}^{2}/2m\to {\left(\hat{p}-\frac{e}{c}A\right)}^{2}/2m$, with A the vector potential of the electromagnetic field and *c* the speed of light. In addition, we need to include a term in the Hamiltonian that corresponds to the interaction energy of an intrinsic magnetic moment with the external magnetic field:

(126) $$H^{\prime }=\sum_{ni\gamma \sigma }2\mu_{B}\left(m+\sigma \right)Ha_{ni\gamma \sigma }^{+}a_{ni\gamma \sigma }^{,}$$

where $\mu_{B}$ is the Bohr magneton, H is the external magnetic field, and σ is the projection of the magnetic moment (spin) onto the direction of the magnetic field, and *m* is the angular momentum quantum number. Since we are only interested in a weak external magnetic field, we only include the spin projection term [in Equation (126)] in the Hamiltonian when a magnetic field is present. By properly treating the local magnetic moment (spin) dependence, we can then compute spin-dependent transport. Note that, since these calculations are approximate, the accuracy is determined both by the strength of the vertex corrections that are ignored and by the smallness of the parameter that governs the cluster expansion.

## 7. Spin-Dependent Transport of Carbon Nanotubes with Chromium Atoms

In this section, we present the application of the above formalism to the problem of chromium impurities doped onto a carbon nanotube. In these final calculations, we neglect the vertex corrections in Equations (60), (61), and (64), and we neglect the static displacements of the atoms. We employ a 2s,2p-states wave-function basis for the neutral carbon atoms and a 3d, 4s-states wave-function basis for neutral chromium atoms. The initial ion core valence of C and Cr atoms $Z^{\lambda_{i}}$ are 4 and 6, respectively. In addition, to reduce the total computational time, we only performed one iteration of the self-consistent iterative procedure. In Equation (16), we used $Z_{i\delta \sigma }^{\lambda }=1$ for the occupied electronic states. The off-diagonal matrix elements of the Hamiltonian in Equation (1) were calculated by including the first three coordination spheres. The Green’s function calculation in Equations (79) and (81) employed 10${}^{3}$ points in the Brillouin zone. All calculations were performed at T=300 K.

**Figure 7:**
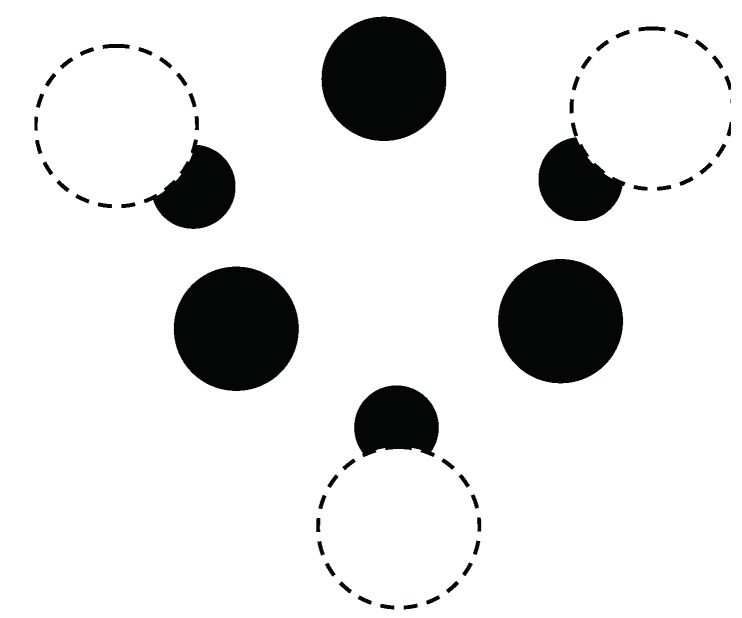
Cross-sectional view of the crystal structure of a (3,0) chiral carbon nanotube with adsorbed Cr atoms. The unit cell of a nanotube is shown. Black solid circles are C atoms, while white dashed-line circles are Cr atoms. The smaller black circles denote Cr atoms located at a large distance from the tube end.

The calculations start with a (3,0) chirality carbon nanotube that is doped with Cr atoms. The geometry for the crystal structure is then optimized by minimizing the free energy *F*, defined in Equation (116). Note that the carbon nanotube (doped with Cr) has a one-dimensional crystal structure. The primitive cell contains 18 nonequivalent atomic positions. Carbon atoms are located at 12 positions on the surface of the inner cylinder. The distance between the carbon atoms is 0.142 nm. Cr atoms are located at the 6 positions on the outer surface of the cylinder opposite the center of a hexagon, the vertices of which are carbon atoms. The distance between carbon atoms and neighboring Cr atoms is 0.22 nm. a cross-sectional view of the crystal structure of the (3,0) chirality carbon nanotube with adsorbed Cr impurities is plotted in Figure 7.

**Figure 8:**
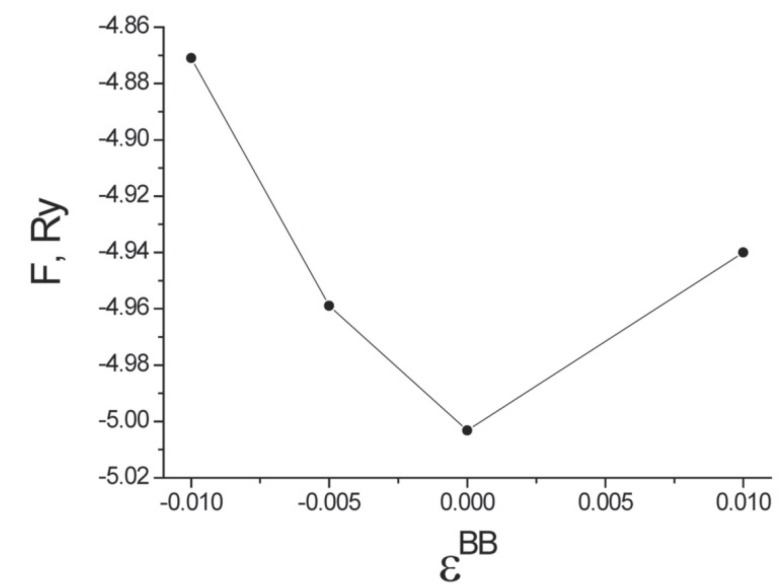
Dependence of the free energy *F* (for carbon nanotubes with five atoms of Cr per primitive unit cell) on the pair correlations of the arrangement of Cr impurities on the lattice sites $\varepsilon^{BB}$.

It turns out that the free energy is minimized by a random arrangement of Cr atoms on the surface of the nanotube. Figure 8 plots the dependence of the free energy on the pair correlations of Cr impurities with $\varepsilon^{BB}=\varepsilon_{lj0i}^{BB}$ in Equation (100) and for the first coordination sphere. The symbol *B* denotes an atom of Cr. The dependence of the free energy on the pair correlations is shown in the region of the free energy minimum.

As shown in Figure 8, the free energy *F* has its minimum at $\varepsilon^{BB}=0$. This implies that the Cr atoms are randomly located on the surface of the carbon nanotube (for this density of impurities). The relative positions of the carbon atoms and the chromium impurities are similar to those found elsewhere for transition-metal dopants on carbon nanotubes using ultrasoft pseudopotentials [40,41]. The value of the localized magneticmoment of a Cr impurity and the induced localized magnetic moment of a C atom in the direction of the magnetic field increases, as expected, with the size of the field. For carbon nanotubes that have five Cr atoms per primitive unit cell, the projection of the Cr magnetic moment varies in the range $m_{Cr}=\left(1.02;2.24\right)\mu_{B}$, while the induced C magnetic moment lies in the range $m_{C}=\left(0.0036;0.02\right)\mu_{B}$ as the external magnetic field increases from zero to H=200 A/m. In this calculation, the magnetic field is oriented along the axis of the carbon nanotube. We also find that the pair correlations for the orientation of the localized magnetic moments in the first coordination sphere satisfies $\varepsilon^{m}=0.235$ when the external field vanishes. This pair correlation nearly vanishes for the second and third coordination spheres. A positive value of $\varepsilon^{m}$ for the first coordination sphere indicates that the induced magnetic moment on a C atom is oriented in the same direction as the magnetic moment of the nearest Cr atom, as one might have expected.

**Figure 9:**
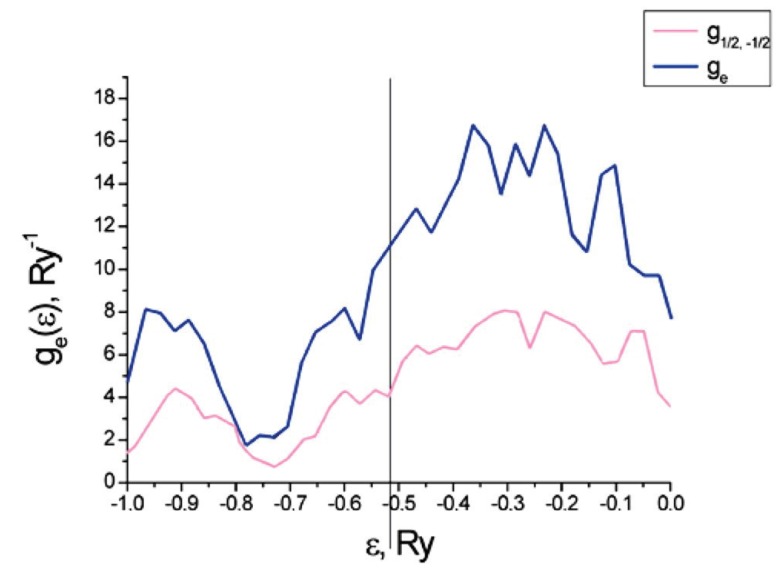
Densities of states of the carbon nanotube with adsorbed Cr.

Figure 9 plots the partial $g_{e\sigma }\left(\varepsilon \right)=\frac{1}{v}\sum_{i,\gamma ,\lambda }P_{0i}^{\lambda }g_{0i\gamma \sigma }^{\lambda }\left(\varepsilon \right)$ and total $g_{e}\left(\varepsilon \right)=\sum_{\sigma }g_{e\sigma }\left(\varepsilon \right)$ densities of states for the electrons on a carbon nanotube with an adsorption of Cr (and vanishing external magnetic field). In this case, we have a paramagnetic phase, so $g_{1/2}\left(\varepsilon \right)=g_{-1/2}\left(\varepsilon \right)$. The vertical line shows the Fermi level $\varepsilon_{F}$.

**Figure 10:**
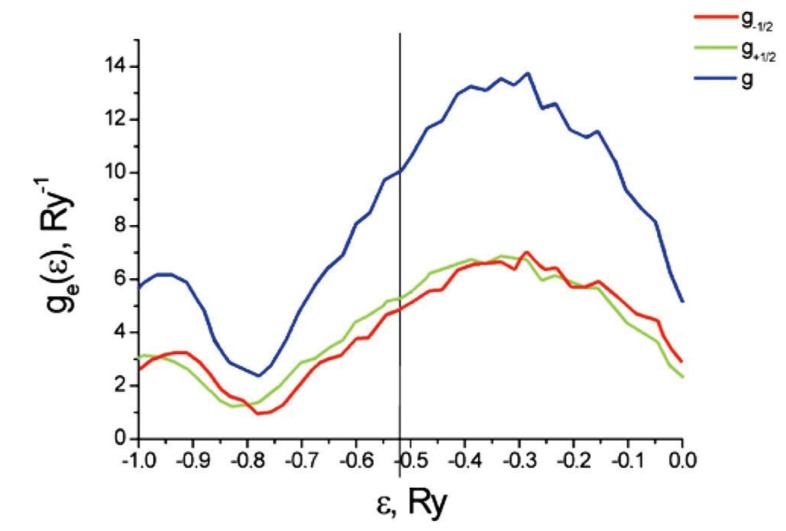
Densities of states for a carbon nanotube with five atoms of Cr per primitive unit cell in external magnetic field of magnitude H=100 A/m and oriented along the tube axis.

Figure 10 plots the partial $g_{e\sigma }\left(\varepsilon \right)$ and total $g_{e}\left(\varepsilon \right)$ densities of states for a carbon nanotube with five atoms of Cr per primitive unit cell and in an external magnetic field of strength H=100 A/m and oriented along the tube axis. The plot is zoomed into the region near the Fermi level.

As shown in Figure 10, the partial density of states $g_{e\sigma }\left(\varepsilon \right)$ for spin σ=1/2 is shifted relative to those for spin σ=−1/2. These results are also qualitatively consistent with results obtained by a different method in [40]. However, the quantitative results differ because this work examines a different chirality for the nanotube than [40] does ((3,0) versus (9,0)).

**Figure 11:**
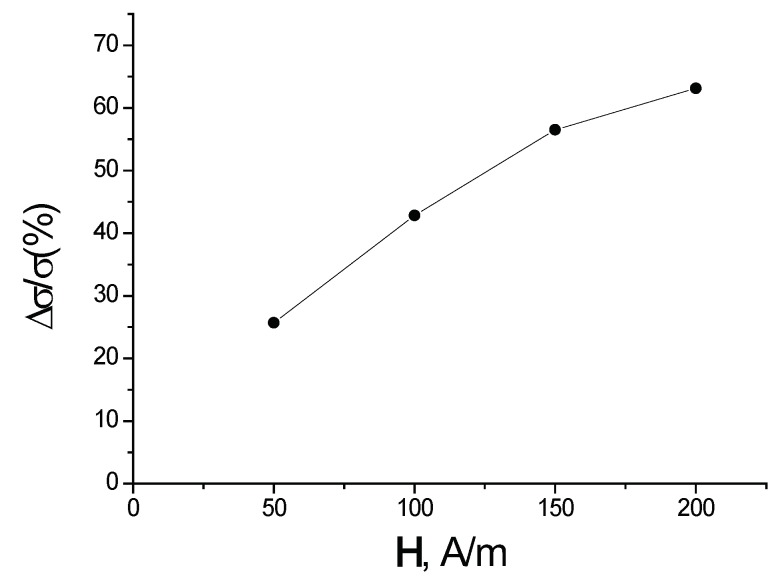
Dependence of the spin polarized electrical conductivity Δσ/σ of a carbon nanotube versus the magnitude of the external magnetic field H.

In Figure 11, the dependence of the spin polarized electrical conductivity $\Delta \sigma /\sigma =(\sigma_{1/2}-\sigma_{-1/2})/\sigma$ of a (3,0) chirality carbon nanotube with five atoms of Cr per primitive unit cell versus the magnitude of the external magnetic field is plotted for T=300 K.

## 8. Conclusions

In this work, previous methods [6,7,8,9,10,13,14,15,16,17,18,19] that describe pure ordered crystals and molecules have been generalized to include disorder effects. The method employed involves a diagrammatic expansion for the electron correlations (under the assumption they are small) along with a cluster-based method to treat the disorder effects (truncated to a small cluster). This method employs Green’s functions but is rooted in density functional theory.

The theory is applied to a particular case of Cr dopants added to a carbon nanotube. In particular, a (3,0) chiral nanotube has five Cr atoms per primitive unit cell added to the system. We find that the resulting spin-dependent electron transport derives from strong electron correlations caused by the presence of chromium atoms. The magnitude of the spin polarized current stems primarily from the difference of the partial densities of states (see Figure 10) with opposite spin projection at the Fermi level. However, it is also affected by the difference between the relaxation times that arise from the different occupation numbers of the single-electron states $Z_{ni\gamma \sigma }^{\lambda }$ of C and Cr (see Equation (95)). The spin polarization of the electric current increases with the concentration of Cr atoms and with the magnitude of the external magnetic field. The results presented here generically agree with those calculated with ultra-soft pseudopotentials [41,42]. The main difference is that, in the present work, we do not find that a gap opens up when the spin polarization becomes large enough.
